# A Natural Xenogeneic Endometrial Extracellular Matrix Hydrogel Toward Improving Current Human *in vitro* Models and Future *in vivo* Applications

**DOI:** 10.3389/fbioe.2021.639688

**Published:** 2021-03-05

**Authors:** Sara López-Martínez, Hannes Campo, Lucía de Miguel-Gómez, Amparo Faus, Alfredo T. Navarro, Ana Díaz, Antonio Pellicer, Hortensia Ferrero, Irene Cervelló

**Affiliations:** ^1^Fundación Instituto Valenciano de Infertilidad, Instituto de Investigación Sanitaria La Fe, Valencia, Spain; ^2^University of Valencia, Valencia, Spain; ^3^IVIRMA Roma, Rome, Italy; ^4^IVIRMA Valencia, Valencia, Spain

**Keywords:** extracellular matrix hydrogels, decellularization, endometrium, 3D culture, tissue engineering

## Abstract

Decellularization techniques support the creation of biocompatible extracellular matrix hydrogels, providing tissue-specific environments for both *in vitro* cell culture and *in vivo* tissue regeneration. We obtained endometrium derived from porcine decellularized uteri to create endometrial extracellular matrix (EndoECM) hydrogels. After decellularization and detergent removal, we investigated the physicochemical features of the EndoECM, including gelation kinetics, ultrastructure, and proteomic profile. The matrisome showed conservation of structural and tissue-specific components with low amounts of immunoreactive molecules. EndoECM supported *in vitro* culture of human endometrial cells in two- and three-dimensional conditions and improved proliferation of endometrial stem cells with respect to collagen and Matrigel. Further, we developed a three-dimensional endometrium-like co-culture system of epithelial and stromal cells from different origins. Endometrial co-cultures remained viable and showed significant remodeling. Finally, EndoECM was injected subcutaneously in immunocompetent mice in a preliminary study to test a possible hypoimmunogenic reaction. Biomimetic endometrial milieus offer new strategies in reproductive techniques and endometrial repair and our findings demonstrate that EndoECM has potential for *in vitro* endometrial culture and as treatment for endometrial pathologies.

## Introduction

The extracellular matrix (ECM) is a complex mixture of fibrous proteins and acts as a physical substrate for cell adhesion, structure, and mechanical stimulation. It also provides biochemical cues for tissue homeostasis, angiogenesis, immune response, and tissue repair (Frantz et al., 2010; Rozario and DeSimone, 2010; Evangelatov and Pankov, 2013; Theocharis et al., 2016; Saldin et al., 2017). Disruption of ECM homeostasis can lead to fibrotic diseases, such as systemic sclerosis, liver cirrhosis, and cardiovascular disease, and can facilitate tumor malignancy and metastatic progression in cancer (Cox and Erler, 2011). The ECM has a unique composition in each tissue including the endometrium, the mucous layer lining the lumen of the uterus and undergoing scar-free remodeling during the menstrual cycle. To achieve successful implantation and placentation, the endometrial ECM is drastically modified along with changes to the luminal epithelium (Guillemot, 1999; Kaloglu and Onarlioglu, 2010; Carrascosa et al., 2018).

The human endometrium consists of an epithelial and a stromal compartment supported by an endogenous niche of stem cells, a vascular compartment, and a population of immune-resident cells (Carrascosa et al., 2018). Three-dimensional models used to recreate the endometrium *in vitro* incorporate ECM-derived matrices such as collagen I and basement membrane extract (Schutte and Taylor, 2012; Asmani et al., 2013; Bayat et al., 2016; Fayazi et al., 2017). However, these standardized matrices do not have the biochemical complexity of the natural endometrial ECM and are not suitable for clinical application because they originate from a tumorigenic cell line.

Decellularization techniques allow the creation of hypoimmunogenic tissue-specific biomaterials, which have a low probability of rejection by the immune system. Among them, ECM hydrogels are used in both three-dimensional culture and for tissue regeneration after injury (Seo et al., 2018; Nehrenheim et al., 2019; Ventura et al., 2020). Hydrogels retain the tissue-specific composition of natural tissues, providing a biomimetic environment that enhances the cellular capacity to migrate, proliferate, differentiate, and mature (Fercana et al., 2017; Link et al., 2017; Viswanath et al., 2017; Sackett et al., 2018; Ventura et al., 2020). Tissue-specific ECM hydrogels have been created for most organ systems and are primarily derived from porcine tissue due to its availability and similarity to humans (Saldin et al., 2017). The development of endometrial tissue-specific ECM hydrogels would be a powerful tool in reproductive medicine for the study of the human endometrium and as a treatment for endometrial pathologies such as Asherman’s syndrome and endometrial atrophy.

We previously reported that tissue-specific ECM-derived coatings obtained from rabbit endometrium at different stages of proliferation influence the *in vitro* growth of rabbit embryos (Campo et al., 2019). In the present study, decellularized endometrium from porcine uterus was used to create an endometrial extracellular matrix (EndoECM) hydrogel. Here, we describe the characterization of the EndoECM at the physical and biochemical levels, comparing its proteomic profile to non-decellularized (No-DC) endometrial hydrogel and myometrial ECM hydrogel controls. We also describe EndoECM biocompatibility and suitability *in vitro*, comparing its potential to standard matrices using human endometrial cells and developing a three-dimensional endometrium-like culture using EndoECM hydrogels with human cells from stem or primary origins. Further, a preliminary test with a subcutaneous murine model was done to determine its *in vivo* applicability. These findings inform the development of EndoECM hydrogels for use in human reproductive medicine.

## Materials and Methods

### Methodology

**FIGURE 1:**
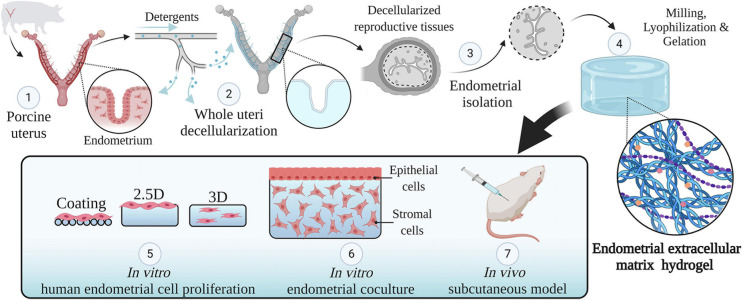
Study design. An overview of the methodology used in this study. Whole porcine uteri were decellularized to obtain acellular tissues (1, 2), the endometrium was then isolated (3) and used to create a hydrogel consisting of solely endometrial ECM (4). The physicochemical features of this hydrogel were analyzed. Different types of endometrial cells were cultured in two- and three-dimensional conditions using endometrial ECM hydrogels, collagen, or Matrigel and their proliferation was compared (5). Next, a three-dimensional endometrium-like co-culture system made of epithelial and stromal cells was developed (6). Finally, the *in vivo* biocompatibility of EndoECM was described in a subcutaneous murine model (7). Image created with BioRender.com.

A detailed description of the methodology used along this research can be found in Figure 1.

### Porcine Uterus Decellularization and Endometrial Isolation

**FIGURE 2:**
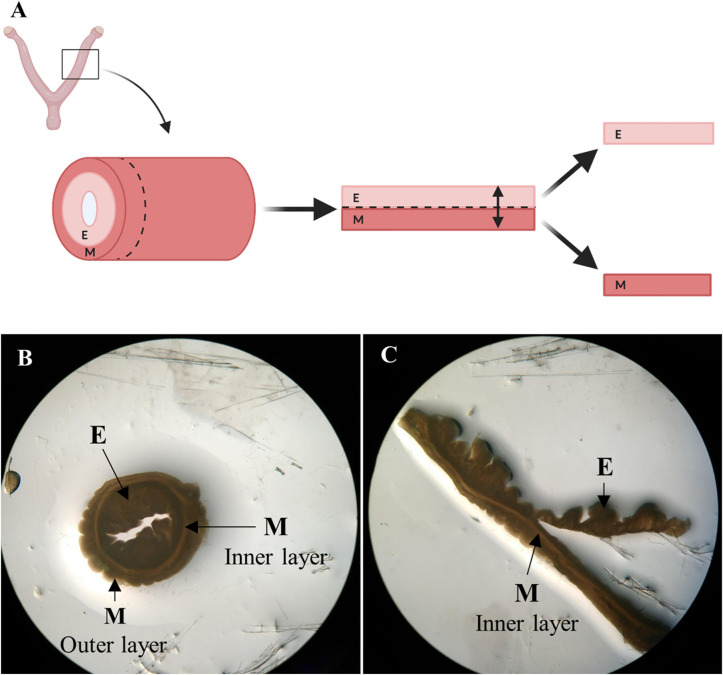
Endometrial isolation by microdissection. **(A)** Schema of manual endometrial isolation. Porcine horns were cut into ring-shaped disks, opened, and cut at the luminal side of the inner circular myometrial layer to isolate the endometrium. Image created with BioRender.com. **(B)** Ring-shaped sections from control uterus showing uterine layers under a stereomicroscope. **(C)** Opened disk from control uterus during the process of microdissection under a stereomicroscope. E: endometrium, M: myometrium.

Entire female porcine reproductive tracts with intact vasculature were donated by a slaughterhouse in Mercavalencia, Spain according to ISO 9001 quality management and subjected to whole organ decellularization. Uterine horns (*n* = 5) were attached to a peristaltic pump (Cole-Parmer Instruments, Fisher Scientific) and decellularized by perfusion of 0.1% sodium dodecyl sulfate (SDS) and 1% Triton X-100 through the uterine artery for 48 h, following a two-cycle protocol (Campo et al., 2017). Then, decellularized (DC) horns stored at −80°C were cut transversally into 1 mm thick ring-shaped disks. The endometrial fraction was isolated via microdissection under a stereomicroscope (SMZ800, Nikon) by cutting the luminal side of the inner circular myometrial layer, which appears as a dense line in both No-DC and DC horns (Figure 2; Campo et al., 2019). The remaining myometrial fraction was kept as a control to verify the correct isolation of pure endometrium and the presence of tissue-specific components during proteomic analysis. Endometrial tissue from No-DC uterine horns (*n* = 5) was also isolated via microdissection as a control for subsequent analyses. Isolated tissues were stored at −80°C.

Additional steps were performed to assure the removal of residual DNA and detergents. Endometrial and myometrial tissue stocks from uterus decellularization were thawed, weighed, and washed in cold PBS (10 mL/g tissue) for 30 min at 200–250 rpm. Then, tissues were incubated for 1 h in 5 μg/mL Dnase I solution (D5025, Sigma-Aldrich) at room temperature and washed again. Aliquots of washing media were stored to detect residual SDS in the DC endometrial tissue.

### SDS Quantification Assay

The presence of SDS in the washing media after endometrial isolation (*n* = 8 DC horn pieces) and subsequent washes (pool of total isolated endometrial tissue) was quantified. It was done by measuring absorbance when SDS reacts with Stains-All dye (Stains-All, Sigma Aldrich; Rupprecht et al., 2015). A calibration curve of 7 standards (0,01–0,1 mg SDS/ml) were made with serial dilutions in PBS. Stains-all was dissolved in N, N-dimethylformamide to 2.0 mg/ml and then diluted 1:20 in ultrapure distilled water to working solution. In the assay, 10 μl of all standards and samples were pipetted in a 96-well plate containing 140 μl 0.1X PBS. Afterward, 50 μl Stains-all working solution (1:20) was added and absorbance was immediately measured at 453 nm using a microplate reader (Spectra Max 190, bioNova Scientífica, S.L.). SDS concentration was calculated by adjusting to the calibration curve with a lineal fit. Total amount of SDS was calculated from initial volume and normalized to individual weight of DC endometrial tissue used per wash. Samples were done in triplicate.

### Histological and Immunostaining Analysis

For every histological and immunohistochemistry/fluorescence analysis, representative tissue sections were deparaffinized using xylene and rehydrated by decreasing concentrations of ethanol and distilled water. Heat-mediated antigen retrieval was performed in 10 mM Citrate Buffer 0.05% tween pH6.0 for 20 min in a 95°C water bath. Sections were permeabilized with 0.05% Tween 1X PBS, blocked with 3–10% bovine serum albumin (BSA) for 1 h at RT and incubated with primary antibodies in 1% BSA overnight at 4°C. For bright-field microscopy, DAB Substrate Kit was used according to the instructions and sections were counterstained by hematoxylin. For immunofluorescence, slides were incubated with an Alexa-Fluor 488 secondary antibody (1:500 dilution) and mounted with 6-diamidino-2-phenylindole (DAPI, P36931, Thermo-Fisher Scientific).

### Decellularization Efficiency and Alpha-Gal Expression

The absence of cellular components and nuclei was investigated by Hematoxylin and Eosin (H&E) staining or by DAPI. Collagen preservation was also evaluated by Masson’s Trichrome (MT) staining using standard protocols. Lastly, samples were immunoassayed for the presence of the alpha-gal epitope (α−Gal Epitope monoclonal antibody M86, ALX-801-090-1, Enzo Life Sciences, 1:5 dilution) by bright-field immunostaining.

### ECM Hydrogel Setup

Isolated DC endometrial tissue stock was flash-frozen in a mortar with liquid N_2_, milled manually, and lyophilized (Lyoquest-85, Telstar, Valencia’s Polytechnic University) over 96 h at 20 Pa. The resulting endometrial lyophilized powder was digested and neutralized using a modified protocol (Brown et al., 2017). Briefly, 1% (w/v) lyophilized powder was suspended in 0.01 M HCl (H1758, Sigma-Aldrich) with 0.1% pepsin (P7000, Sigma-Aldrich) and digested for 48 h under agitation. The solution was left on ice and neutralized with 10% (v/v) 0.1 M NaOH (S8045, Sigma-Aldrich), 11.11% (v/v) 10X PBS (P5493, Sigma-Aldrich), and 1X PBS was used to reach the desire concentration. The resulting EndoECM solution was stored at −80°C.

This process was also performed with isolated DC myometrial and No-DC endometrial tissue stocks to create myometrial extracellular matrix (MyoECM) and No-DC endometrial matrix (No-DC Endo). MyoECM and No-DC Endo were used as controls for subsequent proteomic analyses.

### Whole ECM Characterization

#### DNA Content

DNA was extracted from 23–25 mg wet tissue and 15 mg lyophilized powder using a commercial kit (DNeasy Blood & Tissue, #69504, Qiagen). DNA concentration was measured using the Qubit^TM^ dsDNA HS Assay Kit (Q32851, Thermo-Fisher Scientific) and calculations were conducted normalizing with the initial individual weight. DNA fragmentation was determined using gel electrophoresis and 10 μL of each DNA extracted sample were loaded on a 1% agarose gel RedProtein nucleic acid stain (#41003, Biotium) for a total runtime of 40–50 min at 100 V. A 1 kb plus DNA ladder (#10787018, Invitrogen) was used for comparison.

#### Total Protein Content

Total protein fraction was extracted from 100 mg for wet tissue, 10 mg lyophilized powder, and 35 μL 8 mg/mL EndoECM using 100–400 μL of a modified Laemli buffer (0.125 M Tris HCl, 4% SDS, 0.0001% β-mercaptoethanol; Gibco^TM^ 2-Mercaptoethanol 1000 × 55 mM in DPBS, #21985023, Fisher Scientific) for 48 h at 37°C at 300 rpm. Protein concentration was determined by the Pierce^TM^ BCA protein assay kit (#23225, Thermo-Fisher Scientific) following the standard protocols provided by the manufacturer, all calculations were normalizing using the initial individual weight.

#### Collagen, Glycosaminoglycans, and Elastin Content

Collagen, elastin, and glycosaminoglycans (GAGs) were quantified using Sircol^TM^ insoluble collagen assay, Fastin^TM^ elastin assay, and Blyscan^TM^ glycosaminoglycan assay (Bicolor, Life Sciences Assays), respectively, following the standard protocols provided by the manufacturer. Samples were 23–25 mg for wet tissue, 3–6 mg for lyophilized powder, and 70–250 μL 8 mg/mL EndoECM. Calculations were made normalizing with the initial individual weight.

### Turbidimetric-Kinetic Gelation Assay

The gelation kinetics of EndoECM (*n* = 3) were evaluated by turbidimetry. Absorbance at 405 nm for 100 μL of 3, 6, and 8 mg/mL EndoECM was measured every minute in a microplate reader (SpectraMAX 190, Molecular Devices) at 37°C. Absorbance was normalized with the following formula as described by Freytes et al. (2008):

$$N\,A=\frac{A-A_{0}}{A_{m\,a\,x}-A_{0}}$$

Where NA is the normalized absorbance, A: absorbance t at given time, A_0_: initial absorbance, and Amax: maximum absorbance.

Kinetic parameters (Lag time, Time to half gelation, Time to complete gelation, and Gelation rate) from the different concentrations were compared (Freytes et al., 2008). The Lag time (T_Lag_) was defined as the intercept of the linear region of the gelation curve with 0% absorbance, the Time to half gelation (T_1__/__2_) as the time to 50% absorbance, the Time to complete gelation (T_1_) as the time to 100% absorbance, and the Gelation rate (S) as the slope of the linear region of the gelation curve. Statistical data analyzed respect to concentration of 3 mg/mL.

To test stability and sterility, the resulting EndoECM hydrogels were left in Dulbecco’s modified Eagle’s medium (DMEM/F12; Sigma-Aldrich) containing 10% fetal bovine serum (FBS) and 0.1% streptomycin/penicillin for 7 days under standard *in vitro* culture conditions (37°C, 5% CO_2_).

### Scanning Electron Microscopy

The ultrastructure of 3, 6, and 8 mg/mL EndoECM, 8 mg/mL MyoECM, and 8 mg/mL No-DC Endo hydrogels was evaluated using scanning electron microscopy (SEM). Sample processing was performed in the proteomics facility of SCSIE University of Valencia. This proteomics laboratory is a member of Proteored, PRB3 and is supported by grant PT17/0019, of the PE I + D + i 2013–2016, funded by ISCIII and ERDF. Hydrogels were fixed in 2.5% glutaraldehyde in PBS (Sigma Aldrich, grade II, 25%) for 24 h, washed in PBS, and kept in PBS at 4°C. Then, hydrogels were treated with 2% osmium tetroxide for 2 h and dehydrated in a graded series of alcohol (30, 50, 70, 90, 100% ethanol) for 30 min per wash and kept in 100% ethanol overnight at 4°C. Hydrogels were washed 3 additional times in 100% ethanol for 30 min and critical point dried using a Autosamdri^®^ 814 Critical Point Dryer (Tousimis) with carbon dioxide (CO_2_) at high pressure (1200 pound-force per square inch, psi) as the transitional medium and a maximum heating temperature of 40°C. Dried samples were coated with gold-palladium for 2 min using a SC7640 Sputter Coater (Quorum technologies) and imaged with a SEM FEG Hitachi S-4800 (SCSIE University of Valencia, Spain). To analyze fiber diameter, four measurements per three 30.0 k fields per sample were measured using ImageJ software (Schindelin et al., 2012).

### Proteomic Analysis

The proteomic analysis was performed in the SCSIE proteomics facility of University of Valencia. 50 μg of EndoECM, MyoECM, and No-DC Endo (8 mg/mL) were loaded and resolved in a 1D SDS-PAGE gel. Every sample lane was sliced into seven fragments. Gel slides were digested using sequencing grade trypsin (Promega) at 37°C as described elsewhere (Shevchenko et al., 1996). 200 ng of trypsin were used for samples and digestion was performed at 37°C. Trypsin digestion was stopped with 10% trifluoroacetic acid (TFA) and the supernatant (SN) was removed, then the library gel slides were dehydrated with pure acetonitrile (ACN). The new peptide solutions were combined with the corresponding SN. The peptide mixtures were dried in a speed vacuum and resuspended in 2% ACN; 0.1% TFA. The final volume was between 6 and 25 μL.

Liquid chromatography and tandem mass spectrometry (LC–MS/MS) were performed. 5 μL of sample was loaded onto a trap column (NanoLC Column, 3 μ C18-CL, 350 μmx0.5 mm; Eksigen) and desalted with 0.1% TFA at 2 μL/min for 10 min. Peptides were then loaded onto an analytical column (LC Column, 3 μ C18-CL, 75 μm × 12 cm, Nikkyo) equilibrated in 5% ACN 0.1% FA (formic acid). Elution was carried out with a linear gradient of 5a40% B in A for 60 min. (A: 0.1% FA; B: ACN, 0.1% FA) at a flow rate of 300 nL/min. Peptides were analyzed in a mass spectrometer nanoESI qQTOF (5600 TripleTOF, ABSCIEX). Sample was ionized applying 2.8 kV to the spray emitter. Analysis was carried out in a data-dependent mode. Survey MS1 scans were acquired from 350–1,250 m/z for 250 ms. The quadrupole resolution was set to “UNIT” for MS2 experiments, which were acquired 100–1,500 m/z for 50 ms in “high sensitivity” mode. Following switch criteria were used: charge: 2+ to 5+; minimum intensity; and 70 counts per second (cps). Up to 50 ions were selected for fragmentation after each survey scan. Dynamic exclusion was set to 15 s. The system sensitivity was controlled with 2 fmol of 6 proteins (LC Packings).

ProteinPilot default parameters were used to generate peak list directly from 5600 TripleTof wiff files. The Paragon algorithm (Shilov et al., 2007) of ProteinPilot v 5.0 was used to search the UniprotMammals database (version 03-2018) with the following parameters: Trypsin specificity, (iodoacetamide) cys-alkylation, taxonomy not restricted, and the search effort set to through. The protein grouping was done by Pro group algorithm. The formation of protein groups was guided entirely by observed peptides only, which originated from the experimentally acquired spectra. Because of this, the grouping was guided by spectra. Unobserved regions of protein sequence played no role in explaining the data. Proteins showing unused score > 1.3 were identified with confidence ≥ 95%. Mass spectrometry information of all the fragments were combined for protein identification using the UniprotMammals database (SCSIE University of Valencia).

Filtered output files for each peptide were grouped according to the protein from which they were derived and their individual coverage (% cov) was determined as an indicator of protein abundance of relative quantitation analysis. Common contaminants were excluded following the exclusion criteria of Hodge et al. (2013) and based on their expression in target tissue according to The Human Protein Atlas database^1^ (Uhlen et al., 2015). A list of peptides found in proteomic analysis in EndoECM (Supplementary Table 1), MyoECM (Supplementary Table 2), and No-DC Endo (Supplementary Table 3) can be found in Supplementary Material. Gene ontology (cellular component and molecular function) analysis of the detected proteins was performed through the PANTHER classification system (Mi et al., 2018) and refined according to processes related to ECM.

### *In vitro* Cytocompatibility

This study was approved by the Human Ethics Committee at the IVI Foundation (1706-FIVI-053-IC, Valencia, Spain). *In vitro* cytocompatibility studies were carried out with epithelial and stroma cells from human endometrial stem cell lines and primary human endometrial cells. Epithelial (ICE6) and stromal (ICE7) endometrial stem cell lines were obtained using Hoechst methodology and cloning efficiency (Clone ICE6 and Clone ICE7, Richmond, BC, Canada). Characterization, purity, and clonogenicity were previously reported by Cervelló et al. (2010, 2011). Passages 7 to 12 of ICE6 and ICE7 were used for experiments. Endometrial epithelial cells (EECs) and endometrial stromal cells (ESCs) were obtained from fresh endometrial biopsies from healthy oocyte donors (*n* = 12). Briefly, biopsies were mechanically and enzymatically disaggregated. Then, EECs and ESCs fractions were separated based on size, sedimentation, and membrane filtration (Simón et al., 1993; Cervelló et al., 2010, 2011). Fresh or first passage (P1) EECs and ESCs were used for experiments.

#### Tetrazolium (MTS) Assay

ICE6, ICE7, EECs, and ESCs were cultured in different two- and three-dimensional configurations: on top of a two-dimensional EndoECM coating (2D) or hydrogel (2.5D) and encapsulated within the EndoECM hydrogel (3D) (Link et al., 2017). Two standard culture matrices, type I collagen (collagen solution from bovine skin, C4243, BioReagent) and Matrigel (Corning^®^ Matrigel^®^ Basement Membrane Matrix, 354234, Corning), were used as controls. Acid collagen solution was neutralized with 1% (v/v) 1 M NaOH and 11.11% (v/v) 10X PBS following the manufacturer’s instructions. Collagen and Matrigel were diluted in PBS up to a concentration of 3 mg/mL. For the coating condition, 96-well tissue plates with 20 μL per well of PBS (no-treatment, NoTT), collagen, Matrigel or 3 mg/mL EndoECM were incubated overnight at 4°C. Then, the solution was aspirated and wells were rinsed with PBS. For 2.5D culture, 100 μL of collagen, Matrigel, or 3, 6, or 8 mg/mL EndoECM were incubated at 37°C for 30 min forming a hydrogel. Both conditions were seeded with 15,000 stem cells/cm^2^ or 55,000 primary cells/cm^2^ in 150 μL of culture media (10% FBS-DMEM/F12 medium containing 0.1% antibiotics).

To grow the cells in a 3D environment, they were suspended in ice-cold collagen, Matrigel, or 3, 6, or 8 mg/mL EndoECM (1.0 × 10^6^ cells/mL). Then, 16 μL drops of cell-suspended solution were added per well and incubated at 37°C 30 min before 150 μL of culture media were added to gelled hydrogels.

Viability in 2D and 3D was assessed after 72 h by incubating samples with MTS reagent (CellTiter 96^®^ Cell Proliferation Assay, Promega) for 2 h at 37°C according to the manufacturer’s instructions. Negative controls without cells (blank absorbance values) were included. After incubation, culture media were transferred to a reader plate and absorbance was measured at 490 nm. To determine fold change, data were normalized with the no-treatment for coating and collagen in 2.5D and 3D conditions. Matrix quality among collagen, Matrigel, and EndoECM at 3 mg/mL and EndoECM concentrations was compared.

#### Long-Term Three-Dimensional Co-culture of Endometrial Cells

Endometrial stromal cells (P1) or ICE7 stem cells were mixed with EndoECM (0.75–1.0 × 10^6^ cells/mL) and 150 μL of the mixture was quickly pipetted into a 6.5 mm insert (0.4 μm Pore, Corning Costar Transwell, Sigma-Aldrich) and allowed to solidify. Subsequently, 10% FBS-DMEM/F12 medium containing 0.1% antibiotics was added, and 200,000–300,000 epithelial cells/cm^2^ [EECs (P1) or ICE6] were seeded onto the ESC or ICE7 hydrogel immediately after solidification (Method A) or on day 3 of culture (Method B). Co-cultures were maintained up to 10 days in normoxia (21% O_2_; EECs-ESCs constructs) or hypoxia (2% O_2_; ICE6-7 constructs) under standard cell culture conditions. This protocol is a modification of a previously described protocol for 3D endometrium-like culture systems (Wang et al., 2012).

Construct remodeling was investigated using MT staining. Cell viability was verified using TUNEL assay (DNA Fragmentation Imaging Kit, Roche) for ICE6-7 constructs or *in vivo* live/dead for EECs-ESCs constructs [Invitrogen^TM^ LIVE/DEAD^TM^ cell imaging kit (488/570), Thermo-Fisher Scientific]. Cell proliferation was measured by Ki67 detection (Anti Ki67 polyclonal antibody, ab9260, Sigma-Aldrich, 1:300 dilution). Total cells expressing Ki67 were quantified from three x20 fields per sample using Image ProPlus analysis software v6.3 (MediaCybernetics, Rockville, MD, United States; Francisco et al., 2004). Immunofluorescence was measured for vimentin (Vimentin monoclonal antibody [V9], ab8069, ABCAM, 1:100 dilution) and E-cadherin (E-Cadherin polyclonal antibody, ab53033, ABCAM, 1:100 dilution), specific markers for stroma, and epithelium, respectively.

### *In vivo* Cytocompatibility

All mouse procedures were performed in accordance with Directive 2010/63/EU and the Ethics Committee for Animal Welfare of University of Valencia (A-1510673251016). Injections of 200 μL of 8 mg/mL EndoECM or No-DC Endo (control) were given in the dorsal subcutaneous space of female immunocompetent mice (C57BL/6; UCIM, SCSIE University of Valencia). Hydrogels remained inside the mice for 2 (*n* = 3 for EndoECM and *n* = 2 No-DC Endo), 7 (*n* = 1 EndoECM and *n* = 1 No-DC Endo), and 14 (*n* = 1 for EndoECM and *n* = 1 No-DC Endo) days before mice were euthanized. MT staining was used for quantitative assessment of cell infiltration and scaffold morphology. The macrophage response to implanted hydrogels at 2-, 7-, and 14-days post-surgery was characterized by immunolabeling of CD68 pan-macrophage marker (CD68 polyclonal antibody, ab125212, ABCAM, 1:100 dilution). Five x20 fields per sample were quantify using QuPath analysis software v0.2 (Bankhead et al., 2017).

### Statistical Analysis

Data were analyzed using RStudio^®^ software version 3.6.3 (RStudio Team, 2020) and presented as mean ± standard deviation (SD). All statistical analysis was done with a linear regression model to account for total variability (non-parametric analysis). Here, the value of each variable from the groups of study was estimated by the average difference with respected to a reference group. The *P* value was obtained from contrast hypotheses of the lineal model, indicating with 95% confidence that the difference between the groups is not zero and different without the need of multiple comparison tests. In all cases, *P* < 0.05 was considered significant.

## Results

### Porcine Uterus Decellularization and Endometrial Isolation

**FIGURE 3:**
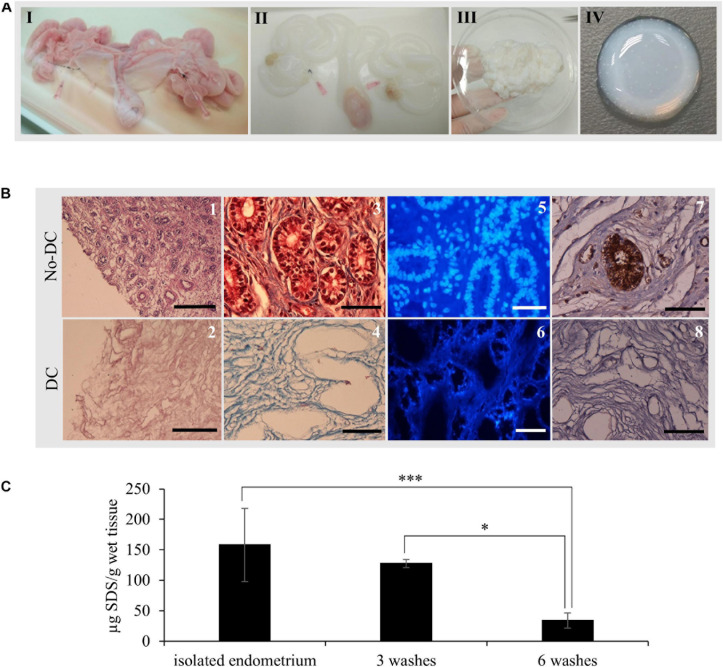
EndoECM hydrogel preparation from porcine uteri. **(A)** uterus before (I) and after (II) decellularization, DC endometrial tissue stock after microdissection (III), and the formed EndoECM hydrogel (IV). **(B)** No-DC endometrial (1, 3, 5, and 7) and DC endometrial tissue (2, 4, 6, and 8). H&E assessment of pure endometrium isolation (1, 2). Scale bars: 250 μm. Analysis of cellular material and collagen composition by trichrome staining (3, 4) and DAPI (5, 6). Immunoreactive porcine α-gal residues (brown) by DAB immunolabeling (7, 8) Scale bars: 50 μm. **(C)** SDS quantification after endometrial isolation, 3 or 6 washes of 30 min with mechanical agitation. *^∗^P* < 0.05 and *^∗∗∗^P* < 0.001.

Whole uterus decellularization was carried out using one or both uterine horns from healthy pigs and the entire endometrial fraction was isolated via microdissection (Figures 2, 3AIII). Decellularization efficiency was verified by H&E (Figures 3B1,2) and MT staining (Figures 3B3,4), which showed complete depletion of cellular material, while DAPI staining showed a total removal of cell nuclei (Figures 3B5,6). Blue coloration in MT staining in DC tissues indicates collagen conservation, a principal ECM component.

Immunostaining of the α-gal epitope, the major responsible for hyperacute rejection of pig xenograft organs in humans (Macher and Galili, 2008), showed a high abundance in luminal and glandular epithelium as well as in blood vessels from No-DC endometrial tissue; in contrast, there was no signal present throughout DC endometrium (Figures 3B7,8).

Quantification of SDS following endometrial isolation detected 158 ± 60.1 μg SDS/g wet tissue. After six 30 min washes with ice-cold PBS under agitation, this was reduced to 33.9 ± 12.4 μg SDS/g wet tissue, corresponding to a statistically significant reduction of 78.6% (*P* < 0.0001; Figure 3C).

### EndoECM Hydrogel Characterization

**FIGURE 4:**
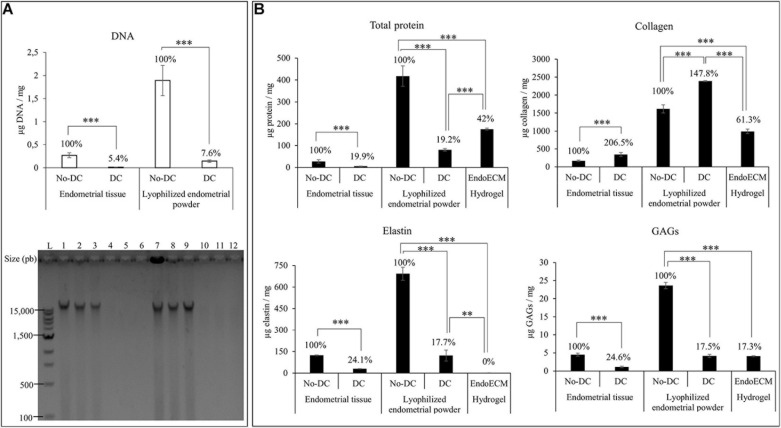
DNA and protein quantification after decellularization, lyophilization, and EndoECM setup. **(A)** DNA quantification and fragment-size analysis in endometrial DC and No-DC wet endometrial tissue and lyophilized endometrial powder. L: ladder; 1,2,3: No-DC wet endometrial tissue replicates; 4,5,6: DC wet endometrial tissue replicates; 7,8,9: No-DC lyophilized endometrial powder replicates; and 10,11,12: DC lyophilized endometrial powder replicates. **(B)** Monitoring of total protein fraction, collagen, elastin, and GAGs in DC and No-DC endometrial tissue, DC and No-DC lyophilized powder, and EndoECM hydrogel. Percentages with respect to endometrial No-DC tissue or lyophilized power. Data in μg/mg. *^∗∗^P* < 0.01, *^∗∗∗^P* < 0.001.

After decellularization, isolated endometrial tissue was milled and lyophilized. Comparison of the DNA content of DC and No-DC showed a significant reduction of nuclear material (5.4 and 7.6% of DNA remained in DC with respect to No-DC wet endometrial tissue and lyophilized endometrial powder, respectively, *P* < 0.0001) and no DNA bands after electrophoretic analysis (Figure 4A). The final viscous solution, EndoECM, formed hydrogels after incubation at 37°C (Figure 3AIV).

Protein quantities and composition were investigated during every step in the production of the EndoECM (shown in Figure 4B). Analysis of total protein content showed a significant 80% decrease in global concentration (19.9 and 19.2% of wet endometrial tissue and lyophilized endometrial powder, respectively) with a significant enrichment of collagen (207 and 148% of wet endometrial tissue and lyophilized endometrial powder, respectively), indicating substantial removal of the cellular protein fraction. Moreover, preservation of elastin and GAGs (25 and 18% of wet endometrial tissue and lyophilized endometrial powder, respectively) was observed (*P* < 0.0001). In EndoECM, the effects of pepsin were apparent, showing an increase in total protein content to 42%, while the percentage of collagens was reduced to 61.3% and no detectable concentration of elastin was found (*P* < 0.0001). Content of GAGs was not affected by pepsin digestion (*P* < 0.0001).

### Gelation Kinetics, Stability, and Ultrastructure

**FIGURE 5:**
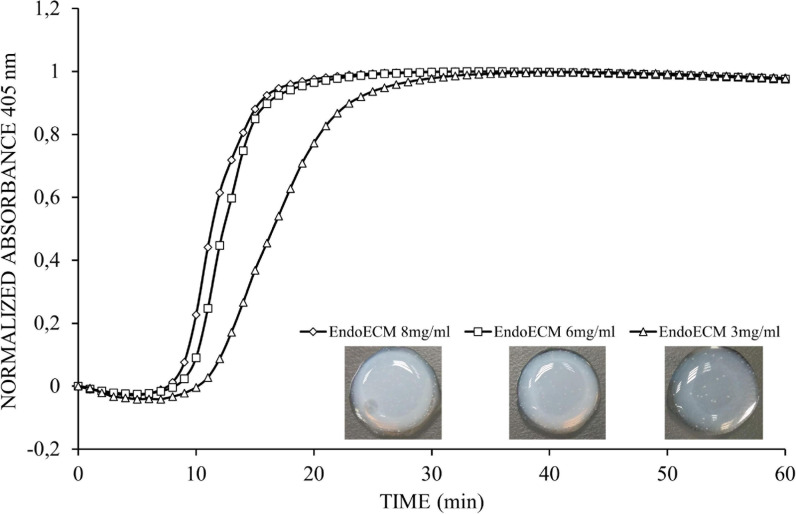
Representative turbidimetric gelation kinetics of EndoECM hydrogels. Comparison of normalized absorbance curves and the metrics analyzed at concentration of 3, 6, and 8 mg/mL.

The gelation kinetics of EndoECM hydrogels from different digestions were evaluated spectrophotometrically. All concentrations presented a sigmoidal curve (Figure 5) with a gelation rate (S) that increased with concentration (0.13 ± 0.03 min^–1^ in 3 mg/mL, 0.22 ± 0.05 min^–1^ in 6 mg/mL, and 0.20 ± 0.02 min^–1^ in 8 mg/mL, *P* < 0.05; Table 1). Time to start of gelation (Lag time, T_Lag_) and the time to 50% (T_1__/__2_) and 100% gelation (T_1_) was inversely related to hydrogel concentration (*P* < 0.05). Hydrogels formed completely after 20 min (20.50 ± 3.81, 14.70 ± 1.12, and 14.10 ± 1.86 in 3, 6, and 8 mg/mL, respectively; Table 1). Opacity and thickness of hydrogels was proportional to ECM concentration (Figure 5). EndoECM hydrogels remained intact and no bacterial growth was seen for 7 days in standard *in vitro* culture conditions, confirming long-term stability, and adequate sterility for culture and subcutaneous injection.

**TABLE 1:** Comparison of turbidimetric metrics at 3, 6, and 8 mg/mL concentration.

Concentration	*T*_Lag_ (min)	*T*_1__/__2_ (min)	*T*_1_ (min)	*S* (min^–1^)
3 mg/ml	12.73 ± 2.00	16.60 ± 2.90	20.48 ± 3.81	0.13 ± 0.03
6 mg/ml	10.09 ± 1.64	12.40 ± 1.27*	14.72 ± 1.12*	0.22 ± 0.05*
8 mg/ml	9.18 ± 1.55*	11.66 ± 1.70*	14.14 ± 1.86*	0.20 ± 0.02*

T_Lag_, Lag; T_1/2_, Time to half gelation; T_1_, time to complete gelation; and S, gelation rate. *P < 0.05.

**FIGURE 6:**
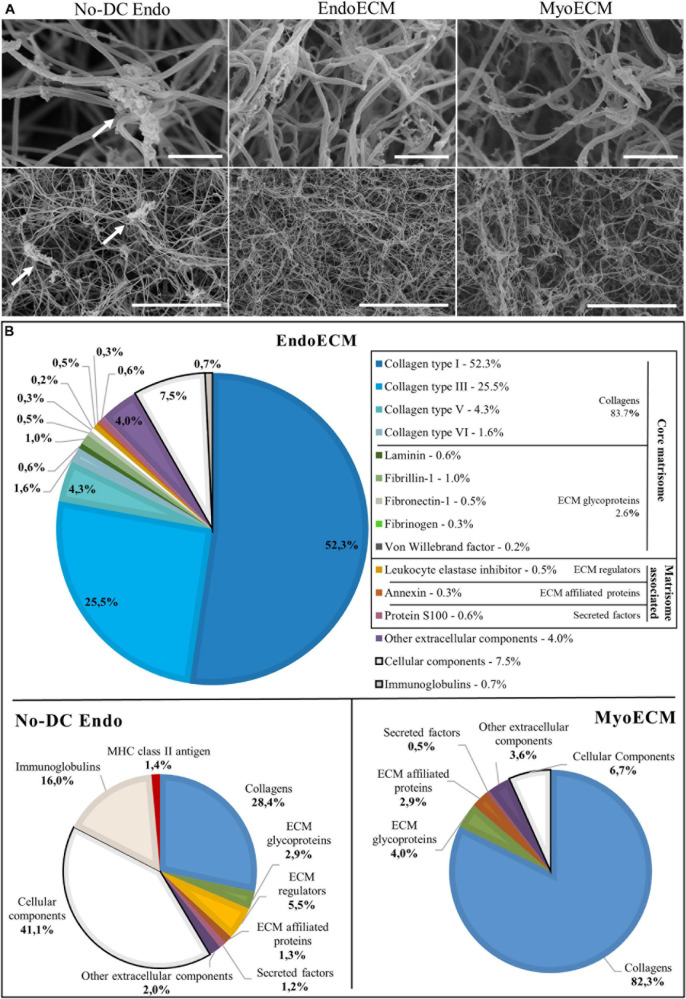
Ultrastructure and proteomic profile of EndoECM hydrogels. **(A)** Scanning electron microscopy images of EndoECM hydrogels in comparison with No-DC Endo and MyoECM hydrogels. Arrows point to remains of cellular components in No-DC Endo. Images at 30.0 k (above) and 5.00 k (below) magnifications. Scale bars are 1.00 μm and 10.0 μm, respectively. **(B)** Quantitative proteomic analysis of EndoECM in comparison with No-DC Endo and MyoECM by LC-MS/MS.

Endometrial extracellular matrix hydrogels present a homogenous, randomly interlocking fibrillar ultrastructure (Figure 6A), and no significant differences in fiber thickness were found among different concentrations (Supplementary Figures 1A,B). Non-decellularized endometrial powder, No-DC Endo, also formed hydrogels and SEM images showed remains of cellular components along the fibers, altering the ultrastructure (Figure 6A, arrows).

Endometrial extracellular matrix hydrogels and MyoECM, both hydrogels made from myometrial fractions, were predominantly composed of approximately 0.10-μm thick fibers, and no significant differences were found (Supplementary Figure 1C). This fiber size and D-period structure are characteristic of collagen fibrils (Veres and Lee, 2012).

### Matrisome of EndoECM Hydrogels

To identify the matrisome, proteins were sorted depending on cellular or extracellular origin and classified according to Matrisome database (MatrisomeDB) into core matrisome proteins (collagens, ECM glycoproteins, and proteoglycans) and matrisome-associated proteins (ECM regulators, ECM-affiliated proteins, and secreted factors). Proteins not found in MatrisomeDB but belonging to the extracellular space were classified as others. Preliminary qualitative analysis showed that half of the No-DC Endo extracellular proteins did not appear in EndoECM. There were four extracellular proteins in EndoECM (dermatopontin, fibrinogen, azurocidin, and extracellular kinases) that did not appear in No-DC Endo (Supplementary Figure 2A and Supplementary Table 4).

Quantitative analysis showed that the ECM hydrogels designed in this study consisted almost entirely of ECM (91.8% in EndoECM and 93.3% in MyoECM), while No-DC Endo comprised 41.4% ECM. These enriched ECM components were principally collagens (83.7% in EndoECM and 82.3% in MyoECM; Figure 6B) and maintained their physiological ratios. Immunoreactive molecules in EndoECM decreased (7.5% cellular components and 0.7% immunoglobulins in EndoECM with respect to 41.1% and 16% in No-DC Endo), while no MHC antigens were detected. No proteoglycans were detected in both EndoECM and No-DC Endo hydrogels.

Moreover, EndoECM and MyoECM hydrogels showed a similar removal of cell immunoreactive components but presented differences in composition in both qualitative (Supplementary Figure 2B and Supplementary Table 4) and quantitative (Figure 6B) analysis.

The role of ECM proteins was identified using GO molecular function and refined according to those functions related to ECM (Supplementary Table 4) in Supplementary Material.

### *In vitro* Cytocompatibility in Two- and Three-Dimensional Cell Culture

**FIGURE 7:**
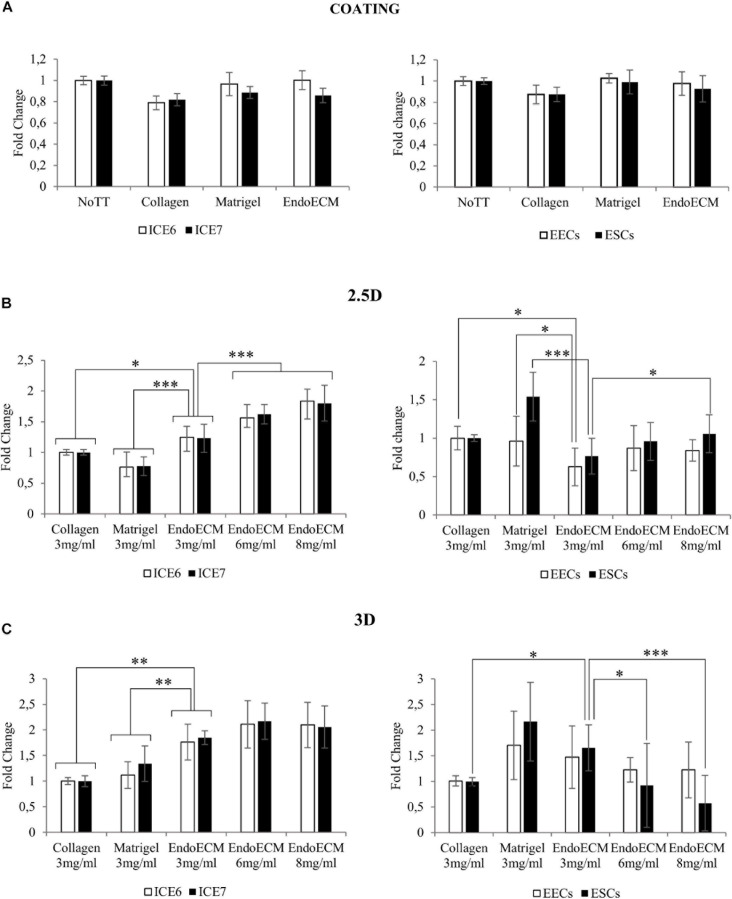
MTS assay of endometrial cells in two- and three-dimensional cell culture. **(A)** Cell proliferation in two-dimensional coating in untreated (NoTT), collagen, Matrigel, or EndoECM coated conditions. **(B)** Cell proliferation in 2.5D culture in collagen, Matrigel, or EndoECM hydrogels at 3 mg/mL. **(C)** Cell proliferation in 3D culture in collagen, Matrigel, and EndoECM hydrogels at concentration of 3 mg/mL. Two extra EndoECM concentrations, 6 and 8 mg/mL, were also tested. Statistical analysis with respect to EndoECM 3 mg/mL. ^∗^*P* < 0.05, ^∗∗^*P* < 0.01, and ^∗∗∗^*P* < 0.001.

Statistical analysis showed no difference in cell growth between uncoated wells and coatings made from collagen, Matrigel, or EndoECM conditions. EndoECM did not induce a significant inhibition of cell growth compared to other groups (Figure 7A).

To test three-dimensional culture cytocompatibility, the matrix quality between collagen, Matrigel, and EndoECM at 3 mg/mL was evaluated. Two extra EndoECM concentrations, 6 and 8 mg/mL, were also included to test the effect of EndoECM concentration. Each cell type was first plated on top of the hydrogels (2.5D culture system; Figure 7B) and a significant increase in proliferation of endometrial stem cells in EndoECM was observed compared to standard matrices at 3 mg/mL. This was not observed in primary endometrial cells, where EECs grown in collagen showed significantly increased proliferation; in Matrigel, both EECs and ESCs showed increased proliferation compared to EndoECM. In ICE6, ICE7, and ESCs, the proliferation in EndoECM increased significantly with concentration (up to 8 mg/mL, *P* < 0.05).

Lastly, each cell type was encapsulated in EndoECM hydrogels to form a 500-μm thick hydrogel (3D culture system; Figure 7C). At 3 mg/mL, a significant increase in proliferation of ICE6, ICE7, and ESCs was observed in EndoECM compared to collagen. Improved proliferation was also observed in EndoECM in relation to Matrigel in endometrial stem cells, but no significant difference was found for EECs or ESCs. ESC proliferation significantly decreased as EndoECM hydrogel concentration increased (*P* < 0.0001 from 3 to 8 mg/mL).

### Long-Term Three-Dimensional Co-Culture of Endometrial Cells

**FIGURE 8:**
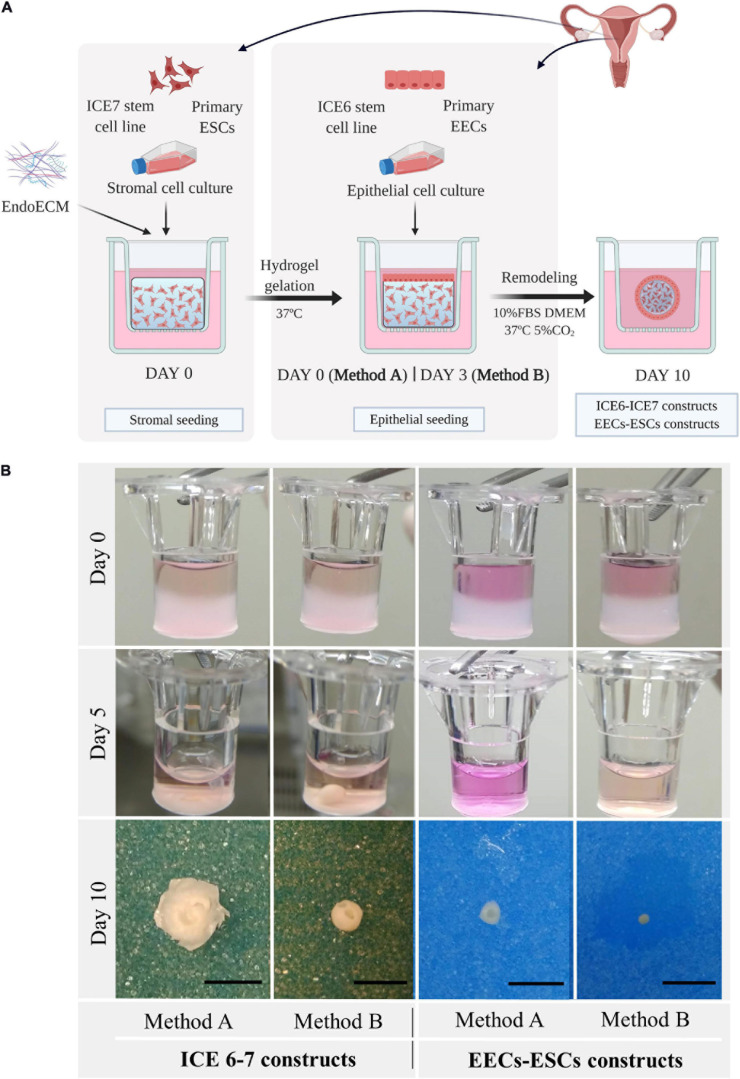
Macroscopic remodeling of *in vitro* endometrium-like culture systems. **(A)** Experimental design. Three-dimensional endometrial-like co-cultures were constructed with primary cells (EEC-ESC constructs) or stem cell lines (ICE6-7 constructs) using EndoECM. Two seeding approaches of EECs, method A or B, were performed to include epithelial and stromal fractions. Picture created with BioRender.com. **(B)** Macroscopic monitoring of ICE6-7 and EECs-ESCs constructs up to 10 days. Scale bars: 50 mm.

Three-dimensional co-culture systems were performed using both stromal and epithelial cells from human endometrial stem cell lines (ICE6-7 constructs) or isolated human primary cells from endometrial biopsies (EECs-ESCs constructs). To slow the reduction of size of the ECM hydrogel, epithelial cells were seeded at day 0 (Method A) or at day 3 (Method B) after stromal cell encapsulation. The ICE6-7 and EECs-ESCs constructs were maintained for up to 10 days. This experimental design is shown in Figure 8A.

After 5 days, all constructs underwent remarkable remodeling, forming a compact round shape at day 7 through day 10 (Figure 8B). When initial cell concentration was high, usually in constructs made using method A, the hydrogel degradation was more aggressive, and constructs acquired a disk shape.

**FIGURE 9:**
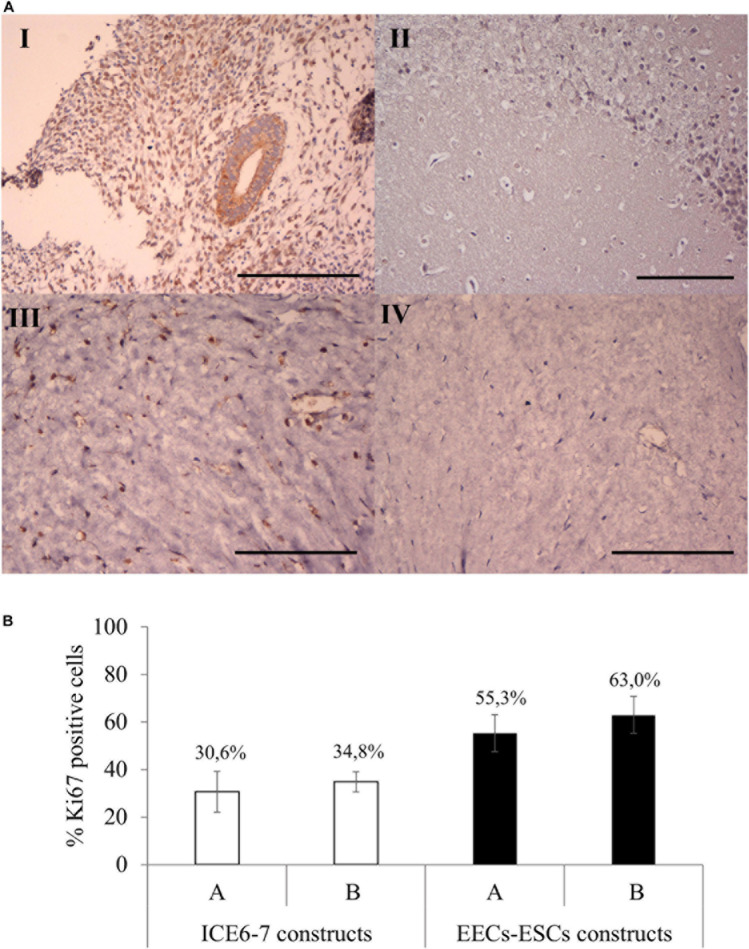
Cell proliferation in long-term *in vitro* endometrium-like co-culture. **(A)** Positive (human endometrium, I) and negative (mouse brain, II) control tissue of Ki67 staining. Positive (III) and negative technical control (no primary antibody, IV) Ki67 staining in cellular constructs at long term. Scale bars: 150 μm. **(B)** Percentage of Ki67 positive cells in ICE6-7 and EECs-ESCs constructs using method A and B at day 10.

Viability assays showed that both ICE6-7 and EECs-ESCs constructs were viable for up to 10 days (around 90%, Supplementary Figure 3). Comparing Ki67 immunohistochemistry, around 33% of cells in the ICE6-7 constructs and 60% in the EECs-ESCs constructs were proliferative, and no statistical difference was found between methods A and B (Figure 9).

**FIGURE 10:**
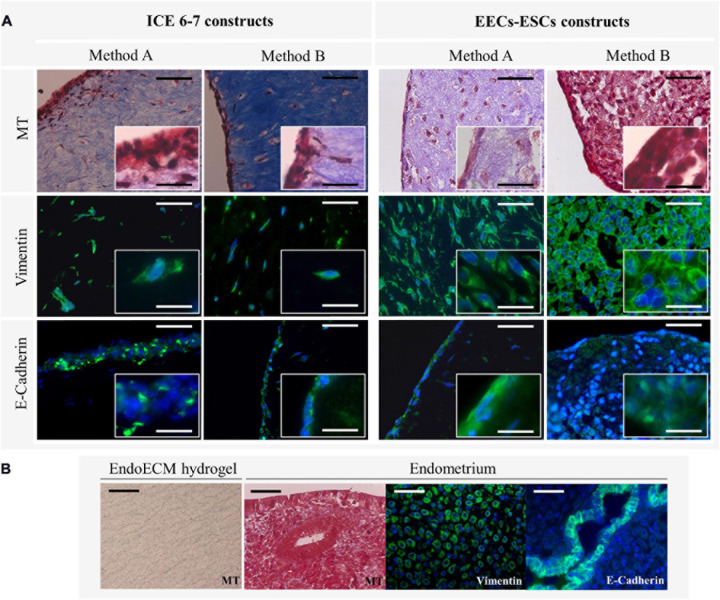
Microscopic remodeling of *in vitro* endometrium-like culture systems. **(A)** Morphological analysis of ICE6-7 and EECs-ESCs constructs at day 10 via MT staining and epithelial (E-cadherin) and stromal (vimentin) markers. Images at 40x and 100x magnifications. Scale bars are 50 μm and 5 μm, respectively. **(B)** Controls of morphological analysis: acellular EndoECM hydrogels and endometrium. Scale bars: 50 μm.

Comparing MT staining, ECM of the contracted constructs were found predominantly in collagens (Figure 10A) at a higher density than in unseeded EndoECM hydrogels (Figure 10B). However, EECs-ESCs constructs made using method B showed little to no blue staining. Both ICE6-7 and EECs-ESCs constructs had vimentin-positive cells surrounded by collagen fibers in a natural 3D shape, while E-cadherin positive epithelial cells formed a luminal-like monolayer (Figure 10A). No apico-basal polarization was seen.

### *In vivo* Immunogenicity

**FIGURE 11:**
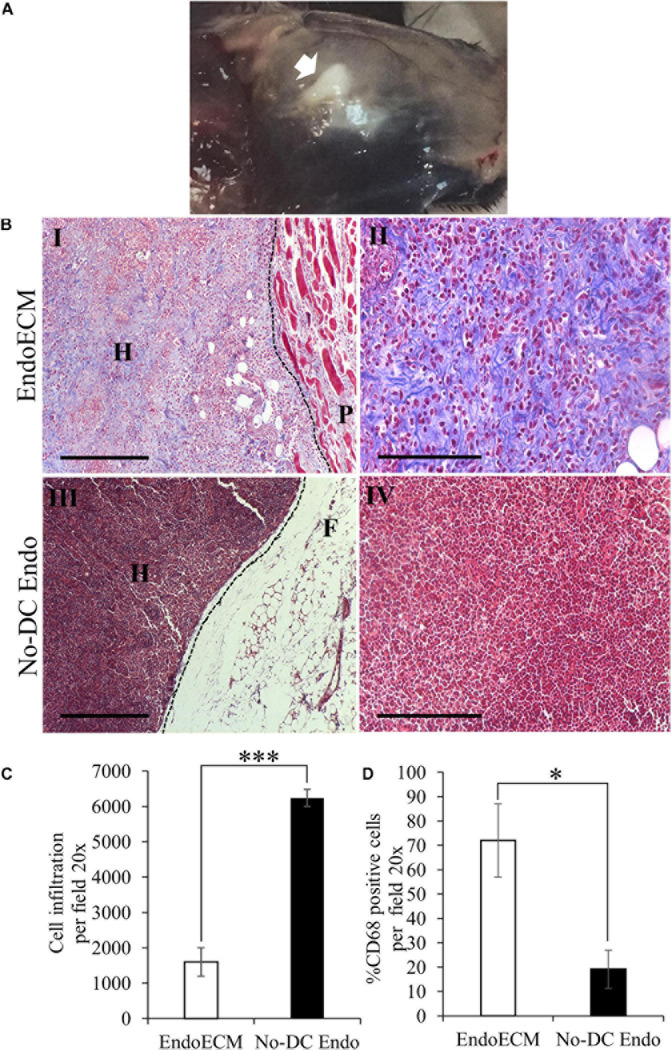
*In vivo* gelation and cytocompatibility of EndoECM hydrogels. **(A)** EndoECM hydrogels after 48 h of subcutaneous injection. **(B)** EndoECM (I, II) and No-DC Endo (III, IV) hydrogels 48 h after subcutaneous injection via MT staining. Dotted lines represent the edge between hydrogels and subcutaneous tissue. H: hydrogel; P: panniculus carnosus. F: subcutaneous fat. Scale bars are 250 μm (I, III) and 150 μm (II, IV). **(C)** Infiltrated inflammatory cells in EndoECM and No-DC Endo hydrogels after 48 h. **(D)** Percentage of infiltrated CD68 positive cells in EndoECM and No-DC Endo hydrogels after 48 h. *^∗^P* < 0.05 and *^∗∗∗^P* < 0.001.

The *in vivo* biocompatibility of EndoECM hydrogels was tested in an immunocompetent murine model. After 48 h, hydrogels appeared gelled and opaque in the subcutaneous tissue (Figure 11A, indicated by an arrow). MT staining showed encapsulation in No-DC Endo and a high infiltration of rounded cells with large nuclei, which corresponds to the morphology of inflammatory cells (Figures 11BIII,IV). There was a 4-fold increase (*P* < 0.0001) in cell infiltration in No-DC Endo (6243 ± 244 cells) compared to EndoECM (1599 ± 402 cells; Figures 11B,C). Further immunological evaluation of CD68 revealed early macrophage infiltration in both EndoECM and No-DC Endo hydrogels that was significantly higher in EndoECM (72.0 ± 15.0% and 19.2 ± 7.77% in EndoECM and No-DC Endo, respectively, *P* < 0.05) at 48 h (Figure 11D). These results provide a proof of concept for our study.

A decrease in volume of both hydrogels was observed after 14 days. Although cell infiltration remained unchanged, MT images of EndoECM samples showed a shift in prevalence from inflammatory-like cells toward spindle-shaped elongated fibroblast-like cells (Supplementary Figures 4A,B). Moreover, the infiltration of CD68 + cells was maintained in EndoECM (72.0 ± 15.0% and 59.5 ± 14.6% at 2 and 14 days, respectively; Supplementary Figures 4C, 5). In No-DC Endo samples, encapsulation disappeared at 14 days, with an increase in CD68 + cells (19.2 ± 7.77% and 35.4 ± 17.5% at 2 and 14 days; Supplementary Figures 4A,C, 5).

## Discussion

The extracellular physicochemical milieu maintains homeostasis within all tissues (Cox and Erler, 2011; Theocharis et al., 2016). Advancements in bioengineering techniques, such as decellularization, permit the generation of natural ECM biomaterials that contain the specific elements of each microenvironment (Loneker et al., 2016; Pouliot et al., 2016; Saldin et al., 2017; Viswanath et al., 2017; Sackett et al., 2018; Nehrenheim et al., 2019). The mammalian ECM is well conserved between species and decellularized xenogeneic tissues are considered biocompatible. Pig tissues are desirable experimental models due to their homogeneity and availability (Seif-Naraghi et al., 2011; Johnson et al., 2014), and porcine decellularized tissues are widely used for clinical applications (Crapo et al., 2011; Saldin et al., 2017; Traverse et al., 2019). However, the potential of these biomaterials for studying drug delivery systems, 3D cultures, tissue implants, and tissue regeneration in reproductive medicine remains largely unexplored.

Here, we describe a novel EndoECM hydrogel of porcine origin. We used a previously published decellularization protocol to isolate pure acellular endometrial tissue from whole uteri (Campo et al., 2017). First, immunocompatibity was assessed by the absence of cells and DNA. A 92.4% reduction of the DNA content was achieved reaching similar levels reported in other publications (Fercana et al., 2017; Sackett et al., 2018; Seo et al., 2018; Bi et al., 2020). Additionally, the α-gal epitope, a carbohydrate responsible for the hyperacute rejection and failure of xenogeneic materials, was absent after decellularization (Macher and Galili, 2008; Pouliot et al., 2016). Then we monitored residual detergents (SDS; Naahidi et al., 2017; Chakraborty et al., 2020) and concentrations that do not interfere with cellular growth were achieved after six washes, similar to the data reported by Cebotari et al. (2010).

Extracellular matrix hydrogel formation is a collagen-based self-assembly process influenced by the natural biochemical composition of the source tissue, including GAGs, proteoglycans, and other ECM proteins (Brightman et al., 2000; Saldin et al., 2017). While no differences in fiber thickness were found between hydrogels, EndoECM gelation kinetics were influenced by ECM concentration, corroborating results from other studies (Wolf et al., 2012). Throughout the generation of the ECM hydrogels, we characterized the different samples (wet tissue, lyophilized powder, and hydrogel). This allowed us to see if important components are enriched or lost during the critical steps of this endometrial ECM hydrogel manufacture process (i.e., lyophilization, ECM digestion). A higher percentage of the total protein concentration in EndoECM with respect to DC lyophilized powder was obtained, likely due to pepsin digestion of DC endometrial tissues. Cryptic ECM peptides liberated by proteases, even in small amounts, exhibit biologically relevant properties such as chemotactic activity and are associated with cell infiltration in both *in vitro* and *in vivo* assays (Agrawal et al., 2011). Even though a reduction in collagen concentration and removal of elastin were observed, the GAGs concentration was not affected. Suggesting the importance of GAGs in the ECM, being key elements in the bioactivity of DC biomaterials implicated in the sequestration and controlled release of growth factors (Mullen et al., 2010; Kowalczewski and Saul, 2018).

We analyzed the EndoECM proteomic profile and compared it to non-decellularized endometrium (No-DC Endo) and the myometrium. Half of the No-DC Endo extracellular proteins did not appear in EndoECM because they were removed. Nevertheless, we detected interesting bioactive ECM proteins shared by both EndoECM and No-DC Endo matrisomes, including fibronectin (which supports initial attachment of endometrial cells; Cook et al., 2017) and Von Willebrand factor (Ishihara et al., 2019). Moreover, we found that certain ECM proteins in EndoECM do not appear in No-DC endo, possibly because they were enriched in EndoECM after decellularization and entered the range of detection. These enriched proteins —dermatopontin (Okamoto and Fujiwara, 2006; Kim et al., 2019), azurocidin (Wiesner and Vilcinskas, 2010; Kasus-Jacobi et al., 2015), fibrinogen (Halper and Kjaer, 2014; Pieters and Wolberg, 2019), and extracellular kinases (Bordoli et al., 2014)— have specific functions in wound healing, chemotaxis, immune response, and antibacterial properties. This suggests that EndoECM preserves the natural ECM composition of the endometrium and has a greater regenerative potential than hydrogels created from non-decellularized endometrium. In addition, the removal of immunoreactive proteins such as immunoglobulins, MHC II, and cellular molecules highlights the low immunoreactive capability of EndoECM. The tissue-specificity related to EndoECM was demonstrated by its unique endometrial protein signatures compared to MyoECM, which also demonstrates the effectiveness of the manual microdissection used in this work. While we consider these proteomic profiles as a baseline, their changes during the porcine estrous cycle can be investigated in future studies.

The main objective of this study was to investigate the potential of this hydrogel as a platform for the *in vitro* culture of the endometrium and assess if we can improve standard techniques by better mimicking the natural endometrial milieu. The previous characterization data suggested that EndoECM could be an improved environment for *in vitro* culture of human endometrial cells. To corroborate this, several endometrial cell types were grown *in vitro* in 2D, 2.5D, and 3D cultures using EndoECM at 3 mg/mL and two standard matrices: collagen and Matrigel. The former was chosen because EndoECM hydrogels exist mainly out of collagen and would give us the opportunity to compare this purified natural material with the tissue-specific mixture. Matrigel, on the other hand, is a popular non-tissue-specific basement membrane preparation rich in ECM components, growth factors and other bioactive proteins (Kleinman et al., 1982, 1986; Vukicevic et al., 1992). No differences were found when EndoECM was used as coatings, confirming cytocompatibility. Instead, we found a significant proliferation increase in EndoECM in 2.5D and 3D systems, mostly in endometrial stem cells, showing a beneficial effect of EndoECM. Nevertheless, even if the improvement of cell growth in ECM hydrogels in three-dimensional cell culture is usually attributed to its tissue-specific properties (Pouliot et al., 2016; Fercana et al., 2017), there is a possibility that other ECM hydrogels yield similar effects. Further studies using non-specific ECM hydrogels could be done to determine this. Likewise, other studies postulate that the differences in cell response are due to mechanical signaling rather than biochemical (Viswanath et al., 2017). The stiffness of substrates also affects stem cell differentiation and proliferation (Engler et al., 2006; Zhao et al., 2014; Gerardo et al., 2019). In sum, both biochemical composition and physical features could play a critical role (Stanton et al., 2019). Future analysis of the stiffness of EndoECM hydrogels will further elucidate the contribution of their physical features in these findings.

When we aimed to engineer the classical structure of the human endometrium by encapsulating stromal cells within EndoECM and covered by epithelial cells, we did not supplement either EndoECM or culture media (i.e., hormones) to examine the inherent impact of the tissue-specific ECM over endometrial cells. Both stem and primary endometrial cells remained viable long-term and rapidly remodeled the EndoECM hydrogels, promoting a reduction in volume. This reduction was the result of the degeneration and contraction of collagen-based ECM hydrogels after interaction with fibroblast-like cells, a common phenomenon observed *in vitro* (Grinnell, 2003). When comparing seeding strategies, method B produced better results than method A. This is probably because of the lower initial cell concentration, resulting in less degradation of the matrix. The rapid remodeling of the ECM hydrogels could present some issues when considering *in vitro* application, reducing the time of exposition of their natural bioactive properties to their environment. In this context, the use of chemical crosslinking (i.e., Genipin; Výborný et al., 2019) or a semi-synthetic mixture with more stable compounds (Valdez et al., 2017; Curley et al., 2019) should be investigated as an alternative. In contrast to previous studies, ICE6-ICE7 and EEC-ESC constructs presented vimentin positive cells with few E-cadherin positive cells on the surface with no apical polarization, likely due to a lack of hormonal stimulation (Wang et al., 2012). Further analysis using cell media combined with hormones could further model epithelial differentiation and organization.

We performed a proof-of-concept experiment to determine whether EndoECM could be used not only as an accurate and novel *in vitro* platform but also as a potential biomaterial for endometrial tissue repair. The *in vivo* biocompatibility of EndoECM hydrogels were preliminary tested in an immunocompetent murine model and the injectability and spontaneous *in vivo* gelation of the endometrial-derived hydrogels were verified by injecting the matrix solution into mouse subcutaneous tissue. At early stages, EndoECM was not encapsulated and a mild infiltration of inflammatory cells was observed accompanied by a significant increase in macrophages in comparison to No-DC Endo. Additionally, a shift in cell infiltration from inflammatory-like cells toward endogenous fibroblast-like cells after 14 days illustrated the *in vivo* repopulation of EndoECM hydrogels (Farnebo et al., 2014). Similar reactions are also found in other subcutaneous biocompatibility studies with decellularized ECM hydrogels (Keane et al., 2013; Farnebo et al., 2014; Porzionato et al., 2015; Wua et al., 2015; Sackett et al., 2018; Zhao et al., 2019). Specifically, a similar infiltration of CD68 macrophages in ECM scaffolds was previously reported (Farnebo et al., 2014; Fercana et al., 2017; Sackett et al., 2018; Seo et al., 2018; Zhao et al., 2019). Macrophages are phagocytic cells that regulate the progression of inflammatory events in tissue repair due to their unique interactions with ECM, where they play a vital role in the correct remodeling of degradable ECM biomaterials (Badylak and Gilbert, 2008; Brown et al., 2012; Scanameo and Ziats, 2019). We also noted a volume reduction of EndoECM over time. The degradation and colonization of the scaffold by the endogenous host cells are important properties for materials with biomedical applications, allowing the reconstitution of native tissue (Wua et al., 2015; Naahidi et al., 2017; dos Santos et al., 2019).

The main biocompatibility concern in decellularized ECM materials is the possible presence of residual detergents, toxins, or cell debris (Lee et al., 2014; Keane et al., 2015; Naahidi et al., 2017; Chakraborty et al., 2020). In this proof of concept, we evaluated the overall acute immune response to EndoECM hydrogels and determined its toxic effects over a relatively short time span. However, many of the immunological mechanisms triggered by EndoECM hydrogels remain unknown. To address these study limitations, an extensive long-term *in vivo* study must be performed to examine the response of EndoECM hydrogels.

In sum, EndoECM hydrogels obtained from porcine DC endometrium are hypoimmunogenic biomaterials containing a reservoir of tissue-specific ECM biomolecules. EndoECM is an optimal matrix for the construction of *in vitro* platforms that closely mimics the native endometrial environment, but it could also treat endometrial pathologies such as Asherman’s syndrome and endometrial atrophy. More investigation is needed to fully develop the possible applications of EndoECM hydrogels in human reproductive medicine.

## Conclusion

We demonstrated an efficient method for the generation of an EndoECM hydrogel of porcine origin, preserving its bioactive and structural components. EndoECM is biocompatible between species supporting *in vitro* growth of different types of human endometrial cells and improving the *in vitro* proliferation rates compared to standard matrices. Furthermore, EndoECM contains a low amount of potentially immunoreactive molecules and preliminary data displays a hypoimmunogenic reaction *in vivo*. Thus, EndoECM is a promising biomaterial mimicking the endometrial milieu and may aid *in vivo* repair of endometrial pathologies.

## Data Availability Statement

The raw data supporting the conclusions of this article will be made available by the authors, without undue reservation.

## Ethics Statement

The studies involving human participants were reviewed and approved by the Human Ethics Committee at the IVI Foundation, Valencia, Spain. The patients/participants provided their written informed consent to participate in this study. The animal study was reviewed and approved by Committee for Animal Welfare of University of Valencia.

## Author Contributions

SL-M: methodology, investigation, data curation, and writing of the original draft. HC: investigation and writing review and editing. LM-G and AF: investigation support. AN: statistical analysis support. AD: licensed veterinarian and animal care support. AP: supervision and funding acquisition. IC: conceptualization, writing review and editing preparation, supervision, project administration, and funding acquisition. All authors contributed to the article and approved the submitted version.

## Conflict of Interest

The authors declare that the research was conducted in the absence of any commercial or financial relationships that could be construed as a potential conflict of interest.

## Footnotes

1 http://www.proteinatlas.org
