# Supplementary material for: A Natural Xenogeneic Endometrial Extracellular Matrix Hydrogel Toward Improving Current Human in vitro Models and Future in vivo Applications
Source: Front Bioeng Biotechnol. 2021 Mar 5;9:639688. doi: 10.3389/fbioe.2021.639688 (PMC7973233; doi:10.3389/fbioe.2021.639688)
Supplement: Supplementary file 1 [file Data_Sheet_1.zip › SUPPLEMENTARY_MATERIALS.pdf]

Supplementary Material

Supplementary Table 1. List of peptides found in proteomic analysis of EndoECM.

General protein classification	Protein classification	Peptide name	% Cov (Coverage)	Accession
Collagen	Collagen type I	Collagen alpha-1(I) chain (Fragments) OS=Cyclopes didactylus OX=84074 GN=COL1A1 PE=1 SV=1	94.9199975	sp|C0HJP1|CO1A1_CYCDI
Collagen	Collagen type I	Collagen alpha-1(I) chain (Fragments) OS=Toxodon sp. OX=1563122 GN=COL1A1 PE=1 SV=1	88.1900012	sp|C0HJP7|CO1A1_TOXSP
Collagen	Collagen type I	Collagen alpha-1(I) chain OS=Dipodomys ordii OX=10020 GN=Col1a1 PE=4 SV=1	64.9100006	tr|A0A1S3GXN3|A0A1S3GXN3_DIPOR
Collagen	Collagen type I	Collagen alpha-1(I) chain OS=Erinaceus europaeus OX=9365 GN=COL1A1 PE=4 SV=1	64.7700012	tr|A0A1S2ZWH5|A0A1S2ZWH5_ERIEU
Collagen	Collagen type I	Collagen alpha-1(I) chain OS=Tarsius syrichta OX=1868482 GN=COL1A1 PE=4 SV=1	54.7200024	tr|A0A1U7U3X6|A0A1U7U3X6_TARSY
Collagen	Collagen type I	Collagen type I alpha 1 chain OS=Myotis lucifugus OX=59463 GN=COL1A1 PE=4 SV=1	65.6400025	tr|G1QDY4|G1QDY4_MYOLU
Collagen	Collagen type I	Collagen type I alpha 1 chain OS=Otolemur garnettii OX=30611 GN=COL1A1 PE=4 SV=1	63.5699987	tr|H0XLS8|H0XLS8_OTOGA
Collagen	Collagen type I	Collagen type I alpha 1 chain OS=Sus scrofa OX=9823 GN=COL1A1 PE=1 SV=1	68.0100024	tr|A0A287A1S6|A0A287A1S6_PIG
Collagen	Collagen type I	Collagen alpha-2(I) chain (Fragments) OS=Orycteropus afer OX=9818 GN=COL1A2 PE=1 SV=1	57.9699993	sp|C0HJN4|CO1A2_ORYAF
Collagen	Collagen type I	Collagen alpha-2(I) chain OS=Bos taurus OX=9913 GN=COL1A2 PE=1 SV=2	61.0000014	sp|P02465|CO1A2_BOVIN
Collagen	Collagen type I	Collagen alpha-2(I) chain OS=Mammut americanum OX=39053 PE=1 SV=3	66.2500024	sp|P85154|CO1A2_MAMAE
Collagen	Collagen type I	Collagen alpha-2(I) chain OS=Pteropus alecto OX=9402 GN=PAL_GLEAN10021742 PE=4 SV=1	43.75	tr|L5KNP2|L5KNP2_PTEAL
Collagen	Collagen type I	Collagen alpha-2(I) chain OS=Rattus norvegicus OX=10116 GN=Col1a2 PE=1 SV=3	49.7099996	tr|F1LS40|F1LS40_RAT
Collagen	Collagen type I	Collagen type I alpha 2 chain OS=Mustela putorius furo OX=9669 GN=COL1A2 PE=4 SV=1	60.2800012	tr|M3XR96|M3XR96_MUSPF
Collagen	Collagen type I	Collagen type I alpha 2 chain OS=Otolemur garnettii OX=30611 GN=COL1A2 PE=4 SV=1	53.0099988	tr|H0WT85|H0WT85_OTOGA
Collagen	Collagen type III	Collagen alpha-1(III) chain OS=Mesocricetus auratus OX=10036 GN=Col3a1 PE=4 SV=1	45.5300003	tr|A0A1U7QZS1|A0A1U7QZS1_MESAU
Collagen	Collagen type III	Collagen type III alpha 1 chain OS=Cavia porcellus OX=10141 GN=COL3A1 PE=4 SV=2	49.5200008	tr|H0V8P9|H0V8P9_CAVPO
Collagen	Collagen type III	Collagen type III alpha 1 chain OS=Felis catus OX=9685 GN=COL3A1 PE=4 SV=2	52.2199988	tr|M3WL90|M3WL90_FELCA
Collagen	Collagen type III	Collagen type III alpha 1 chain OS=Ictidomys tridecemlineatus OX=43179 GN=COL3A1 PE=4 SV=1	44.3199992	tr|A0A287DCB4|A0A287DCB4_ICTTR
Collagen	Collagen type III	Collagen type III alpha 1 chain OS=Loxodonta africana OX=9785 GN=COL3A1 PE=4 SV=1	38.1000012	tr|G3TH25|G3TH25_LOXAF
Collagen	Collagen type III	Collagen type III alpha 1 chain OS=Myotis lucifugus OX=59463 GN=COL3A1 PE=4 SV=1	41.2800014	tr|G1PR85|G1PR85_MYOLU
Collagen	Collagen type III	Collagen type III alpha 1 chain OS=Oryctolagus cuniculus OX=9986 GN=COL3A1 PE=4 SV=1	50.1999974	tr|G1T8J0|G1T8J0_RABIT
Collagen	Collagen type III	Collagen type III alpha 1 chain OS=Ovis aries OX=9940 GN=COL3A1 PE=4 SV=1	50.7200003	tr|W5Q4S0|W5Q4S0_SHEEP
Collagen	Collagen type III	Collagen, type III, alpha 1 OS=Bos taurus OX=9913 GN=COL3A1 PE=2 SV=1	49.9300003	tr|Q08E14|Q08E14_BOVIN
Collagen	Collagen type III	REVERSED Collagen type III alpha 1 chain OS=Ailuropoda melanoleuca OX=9646 GN=COL3A1 PE=4 SV=1	44.9499995	RRRRRtr|G1LYT1|G1LYT1_AILME
Collagen	Collagen type V	ProCollagen alpha 1(V) OS=Sus scrofa OX=9823 GN=COL5A1 PE=2 SV=1	13.0400002	tr|Q59IP3|Q59IP3_PIG
Collagen	Collagen type V	ProCollagen alpha 2(V) OS=Sus scrofa OX=9823 GN=COL5A2 PE=2 SV=1	38.1599993	tr|Q59IP2|Q59IP2_PIG
Collagen	Collagen type V	Collagen type V alpha 2 chain OS=Sarcophilus harrisii OX=9305 GN=COL5A2 PE=4 SV=1	26.5300006	tr|G3VWK0|G3VWK0_SARHA
Collagen	Collagen type VI	Collagen alpha-2(VI) chain OS=Fukomys damarensis OX=885580 GN=H920_10447 PE=4 SV=1	12.7399996	tr|A0A091DC51|A0A091DC51_FUKDA
Collagen	Collagen type VI	Collagen type VI alpha 3 chain OS=Otolemur garnettii OX=30611 GN=COL6A3 PE=4 SV=1	3.02799996	tr|H0XEJ5|H0XEJ5_OTOGA
Collagen	Collagen type VI	Collagen type VI alpha 3 chain OS=Sus scrofa OX=9823 GN=COL6A3 PE=1 SV=1	7.11300001	tr|A0A287BLM4|A0A287BLM4_PIG

Collagen	Collagen type VI	Collagen, type VI, alpha 3 OS=Mus musculus OX=10090 GN=Col6a3 PE=1 SV=2	5.54200001	tr|E9PWQ3|E9PWQ3_MOUSE
ECM glycoproteins	Laminin	Laminin subunit beta 2 OS=Felis catus OX=9685 GN=LAMB2 PE=4 SV=2	7.10299984	tr|M3WC88|M3WC88_FELCA
ECM glycoproteins	Laminin	Laminin subunit alpha 5 OS=Sus scrofa OX=9823 GN=LAMA5 PE=4 SV=1	1.822	tr|A0A287AEH1|A0A287AEH1_PIG
ECM glycoproteins	Laminin	Laminin, alpha 5 OS=Pan troglodytes OX=9598 GN=LAMA5 PE=2 SV=1	1.75899994	tr|K7D2I3|K7D2I3_PANTR
ECM glycoproteins	Fibrillin	Fibrillin-1 OS=Sus scrofa OX=9823 GN=FBN1 PE=1 SV=3	18.8800007	tr|F1SN67|F1SN67_PIG
ECM glycoproteins	Fibronectin 1	Fibronectin 1 OS=Sus scrofa OX=9823 GN=FN1 PE=1 SV=1	9.16000009	tr|A0A286ZY95|A0A286ZY95_PIG
ECM glycoproteins	Fibrinogen	Fibrinogen beta chain OS=Sus scrofa OX=9823 GN=FGB PE=1 SV=2	4.62599993	tr|I3L651|I3L651_PIG
ECM glycoproteins	von Willebrand factor	von Willebrand factor OS=Sus scrofa OX=9823 GN=VWF PE=1 SV=2	2.77900007	tr|K7GNN0|K7GNN0_PIG
ECM regulators	Leukocyte elastase inhibitor	Leukocyte elastase inhibitor OS=Sus scrofa OX=9823 GN=SERPINB1 PE=1 SV=1	10.0500003	tr|F2Z5B1|F2Z5B1_PIG
ECM affiliated proteins	Annexin	Annexin OS=Pteropus alecto OX=9402 GN=PAL_GLEAN10023415 PE=3 SV=1	6.15300015	tr|L5K0Z3|L5K0Z3_PTEAL
Secreted factors	Protein S100	Protein S100 OS=Pan troglodytes OX=9598 GN=S100A8 PE=3 SV=1	11.8299998	tr|H2Q028|H2Q028_PANTR
Others extracellular components	Azurocidin	Azurocidin OS=Sus scrofa OX=9823 GN=AZU1 PE=1 SV=2	51.6300023	sp|P80015|CAP7_PIG
Others extracellular components	Dermatopontin	Dermatopontin OS=Ovis aries OX=9940 GN=DPT PE=4 SV=1	11.9400002	tr|W5PHI8|W5PHI8_SHEEP
Others extracellular components	extracellular tyrosine-protein kinase	extracellular tyrosine-protein kinase PKDCC OS=Dipodomys ordii OX=10020 GN=Pkdcc PE=4 SV=1	2.67399997	tr|A0A1S3F9Z3|A0A1S3F9Z3_DIPOR
Others extracellular components	Serum albumin	Serum albumin OS=Homo sapiens OX=9606 PE=2 SV=1	7.38900006	tr|Q56G89|Q56G89_HUMAN
Cellular components	Beta actin	Beta actin OS=Cricetidae sp. OX=36483 PE=2 SV=1	22.6699993	tr|O35247|O35247_CRISP
Cellular components	Caveolin	Caveolin OS=Myotis davidii OX=225400 GN=MDA_GLEAN10003317 PE=3 SV=1	14.61	tr|L5LJI4|L5LJI4_MYODS
Cellular components	Caveolin	Caveolin OS=Sus scrofa OX=9823 GN=CAV2 PE=2 SV=1	17.2800004	tr|G8GCE6|G8GCE6_PIG
Cellular components	cellular enzymes	Glyceraldehyde-3-phosphate dehydrogenase OS=Gammaproteobacteria bacterium OX=1913989 GN=gap PE=3 SV=1	6.45200014	tr|A0A2G6KYX1|A0A2G6KYX1_9GAMM
Cellular components	cellular enzymes	Glyceraldehyde-3-phosphate dehydrogenase OS=Homo sapiens OX=9606 GN=HEL-S-162eP PE=2 SV=1	10.4500003	tr|V9HVZ4|V9HVZ4_HUMAN
Cellular components	cellular enzymes	GMP synthase [glutamine-hydrolyzing] OS=Deltaproteobacteria bacterium OX=2026735 GN=guaA PE=3 SV=1	3.92899998	tr|A0A2G6NF56|A0A2G6NF56_9DELT
Cellular components	Junction plakoglobin	Junction plakoglobin OS=Ailuropoda melanoleuca OX=9646 GN=JUP PE=4 SV=1	26.96	tr|G1LGG3|G1LGG3_AILME
Cellular components	Heat shock protein	Epididymis secretory protein Li 102 OS=Homo sapiens OX=9606 GN=HEL-S-102 PE=2 SV=1	34.6300006	tr|V9HW43|V9HW43_HUMAN
Ig	IgG	IgG heavy chain OS=Sus scrofa OX=9823 GN=IGHG PE=2 SV=1	13.6500001	tr|L8B0U3|L8B0U3_PIG

Supplementary Table 2. List of peptides found in proteomic analysis of MyoECM.

General protein classification	Protein classification	Peptide name	% Cov (Coverage)	Accession
Collagen	Collagen type I	Collagen alpha-1(I) chain (Fragments) OS=Cyclopes didactylus OX=84074 GN=COL1A1 PE=1 SV=1	91.50000215	sp|C0HJP1|CO1A1_CYCDI
Collagen	Collagen type I	Collagen alpha-1(I) chain (Fragments) OS=Equus sp. OX=46122 GN=COL1A1 PE=1 SV=1	78.21000218	sp|C0HJN9|CO1A1_EQUSP
Collagen	Collagen type I	Collagen alpha-1(I) chain OS=Castor canadensis OX=51338 GN=COL1A1 PE=4 SV=1	59.85999703	tr|A0A250Y7T0|A0A250Y7T0_CASCN
Collagen	Collagen type I	Collagen alpha-1(I) chain OS=Dipodomys ordii OX=10020 GN=Col1a1 PE=4 SV=1	63.8899982	tr|A0A1S3GXN3|A0A1S3GXN3_DIPOR
Collagen	Collagen type I	Collagen alpha-1(I) chain OS=Fukomys damarensis OX=885580 GN=H920_03660 PE=4 SV=1	41.76999927	tr|A0A091DWW2|A0A091DWW2_FUKDA
Collagen	Collagen type I	Collagen type I alpha 1 chain OS=Chlorocebus sabaeus OX=60711 GN=COL1A1 PE=4 SV=1	64.55000043	tr|A0A0D9QYW4|A0A0D9QYW4_CHLSB
Collagen	Collagen type I	Collagen type I alpha 1 chain OS=Felis catus OX=9685 GN=COL1A1 PE=4 SV=2	63.05999756	tr|M3W2F5|M3W2F5_FELCA

Collagen	Collagen type I	Collagen type I alpha 1 chain OS=Gorilla gorilla gorilla OX=9595 GN=COL1A1 PE=4 SV=1	65.03000259	tr|G3RBN8|G3RBN8_GORGO
Collagen	Collagen type I	Collagen type I alpha 1 chain OS=Monodelphis domestica OX=13616 GN=COL1A1 PE=4 SV=2	60.40999889	tr|F7CV32|F7CV32_MONDO
Collagen	Collagen type I	Collagen type I alpha 1 chain OS=Myotis lucifugus OX=59463 GN=COL1A1 PE=4 SV=1	63.92999887	tr|G1QDY4|G1QDY4_MYOLU
Collagen	Collagen type I	Collagen type I alpha 1 chain OS=Ornithorhynchus anatinus OX=9258 GN=COL1A1 PE=4 SV=1	43.88999939	tr|F7ESN3|F7ESN3_ORNAN
Collagen	Collagen type I	Collagen type I alpha 1 chain OS=Ovis aries OX=9940 GN=COL1A1 PE=4 SV=1	61.73999906	tr|W5P481|W5P481_SHEEP
Collagen	Collagen type I	Collagen type I alpha 1 chain OS=Sus scrofa OX=9823 GN=COL1A1 PE=1 SV=1	70.87000012	tr|A0A287A1S6|A0A287A1S6_PIG
Collagen	Collagen type I	Collagen type I alpha 2 chain OS=Felis catus OX=9685 GN=COL1A2 PE=4 SV=2	63.59000206	tr|M3WVN3|M3WVN3_FELCA
Collagen	Collagen type I	Collagen type I alpha 2 chain OS=Loxodonta africana OX=9785 GN=COL1A2 PE=4 SV=1	49.48999882	tr|G3TIC0|G3TIC0_LOXAF
Collagen	Collagen type I	Collagen type I alpha 2 chain OS=Mustela putorius furo OX=9669 GN=COL1A2 PE=4 SV=1	58.74000192	tr|M3XR96|M3XR96_MUSPF
Collagen	Collagen type I	Collagen type I alpha 2 chain OS=Otolemur garnettii OX=30611 GN=COL1A2 PE=4 SV=1	49.77999926	tr|H0WT85|H0WT85_OTOGA
Collagen	Collagen type I	Collagen alpha-2(I) chain (Fragments) OS=Equus sp. OX=46122 GN=COL1A2 PE=1 SV=1	79.71000075	sp|C0HJP0|CO1A2_EQUSP
Collagen	Collagen type I	Collagen alpha-2(I) chain (Fragments) OS=Orycteropus afer OX=9818 GN=COL1A2 PE=1 SV=1	69.42999959	sp|C0HJN4|CO1A2_ORYAF
Collagen	Collagen type I	Collagen alpha-2(I) chain (Fragments) OS=Toxodon sp. OX=1563122 GN=COL1A2 PE=1 SV=1	81.83000088	sp|C0HJP8|CO1A2_TOXSP
Collagen	Collagen type I	Collagen alpha-2(I) chain OS=Bos taurus OX=9913 GN=COL1A2 PE=1 SV=2	59.68000293	sp|P02465|CO1A2_BOVIN
Collagen	Collagen type I	Collagen alpha-2(I) chain OS=Canis lupus familiaris OX=9615 GN=COL1A2 PE=4 SV=1	58.49000216	tr|F1PHY1|F1PHY1_CANLF
Collagen	Collagen type I	Collagen alpha-2(I) chain OS=Oryctolagus cuniculus OX=9986 GN=COL1A2 PE=4 SV=1	47.06999958	tr|G1T2Z5|G1T2Z5_RABIT
Collagen	Collagen type I	Collagen alpha-2(I) chain OS=Pteropus alecto OX=9402 GN=PAL_GLEAN10021742 PE=4 SV=1	43.45999956	tr|L5KNP2|L5KNP2_PTEAL
Collagen	Collagen type I	Collagen alpha-2(I) chain OS=Rattus norvegicus OX=10116 GN=Col1a2 PE=1 SV=3	45.91999948	tr|F1LS40|F1LS40_RAT
Collagen	Collagen type III	Collagen type III alpha 1 chain OS=Ailuropoda melanoleuca OX=9646 GN=COL3A1 PE=4 SV=1	43.09999943	tr|G1LYT1|G1LYT1_AILME
Collagen	Collagen type III	Collagen type III alpha 1 chain OS=Equus caballus OX=9796 GN=COL3A1 PE=4 SV=1	39.75999951	tr|F6R528|F6R528_HORSE
Collagen	Collagen type III	Collagen type III alpha 1 chain OS=Felis catus OX=9685 GN=COL3A1 PE=4 SV=2	53.04999948	tr|M3WL90|M3WL90_FELCA
Collagen	Collagen type III	Collagen type III alpha 1 chain OS=Ictidomys tridecemlineatus OX=43179 GN=COL3A1 PE=4 SV=1	40.63000083	tr|A0A287DCB4|A0A287DCB4_ICTTR
Collagen	Collagen type III	Collagen type III alpha 1 chain OS=Myotis lucifugus OX=59463 GN=COL3A1 PE=4 SV=1	39.84999955	tr|G1PR85|G1PR85_MYOLU
Collagen	Collagen type V	ProCollagen alpha 2(V) OS=Sus scrofa OX=9823 GN=COL5A2 PE=2 SV=1	19.81000006	tr|Q59IP2|Q59IP2_PIG
Collagen	Collagen type V	Collagen type V alpha 1 chain OS=Ailuropoda melanoleuca OX=9646 GN=COL5A1 PE=4 SV=1	30.82999885	tr|G1LX86|G1LX86_AILME
Collagen	Collagen type V	Collagen type V alpha 2 chain OS=Sarcophilus harrisii OX=9305 GN=COL5A2 PE=4 SV=1	23.92999977	tr|G3VWK0|G3VWK0_SARHA
Collagen	Collagen type VI	Collagen type VI alpha 1 chain OS=Mustela putorius furo OX=9669 GN=COL6A1 PE=4 SV=1	10.32999977	tr|M3XRA5|M3XRA5_MUSPF
Collagen	Collagen type VI	Collagen type VI alpha 2 chain OS=Ailuropoda melanoleuca OX=9646 GN=COL6A2 PE=4 SV=1	18.73999983	tr|G1L445|G1L445_AILME
Collagen	Collagen type VI	Collagen alpha-2(VI) chain OS=Bos mutus OX=72004 GN=M91_02728 PE=4 SV=1	25.29000044	tr|L8I5Z3|L8I5Z3_9CETA
Collagen	Collagen type VI	Collagen type VI alpha 3 chain OS=Gorilla gorilla gorilla OX=9595 GN=COL6A3 PE=4 SV=2	8.90500024	tr|G3S5Z6|G3S5Z6_GORGO
Collagen	Collagen type VI	Collagen type VI alpha 3 chain OS=Otolemur garnettii OX=30611 GN=COL6A3 PE=4 SV=1	6.593000144	tr|H0XEJ5|H0XEJ5_OTOGA
Collagen	Collagen type VI	Collagen type VI alpha 3 chain OS=Ovis aries OX=9940 GN=COL6A3 PE=4 SV=1	8.370000124	tr|W5QCP9|W5QCP9_SHEEP
Collagen	Collagen type VI	Collagen type VI alpha 3 chain OS=Sus scrofa OX=9823 GN=COL6A3 PE=1 SV=1	13.33000064	tr|A0A287BLM4|A0A287BLM4_PIG
Collagen	Collagen type XII	Collagen type XII alpha 1 chain OS=Ovis aries OX=9940 GN=COL12A1 PE=4 SV=1	3.392000124	tr|W5P8W6|W5P8W6_SHEEP
ECM glycoproteins	Laminin	Laminin subunit alpha 5 OS=Sus scrofa OX=9823 GN=LAMA5 PE=4 SV=1	3.616999835	tr|A0A287AEH1|A0A287AEH1_PIG
ECM glycoproteins	Laminin	Laminin, alpha 5 OS=Pan troglodytes OX=9598 GN=LAMA5 PE=2 SV=1	2.490000054	tr|K7D2I3|K7D2I3_PANTR
ECM glycoproteins	Laminin	Laminin subunit beta 2 OS=Sus scrofa OX=9823 GN=LAMB2 PE=1 SV=3	12.98999935	tr|F1SPT5|F1SPT5_PIG
ECM glycoproteins	Fibrillin	Fibrillin-1 OS=Sus scrofa OX=9823 GN=FBN1 PE=1 SV=3	20.64999938	tr|F1SN67|F1SN67_PIG
ECM glycoproteins	Fibrillin	fibrillin-2 OS=Erinaceus europaeus OX=9365 GN=FBN2 PE=4 SV=1	7.417999953	tr|A0A1S3A1M5|A0A1S3A1M5_ERIEU
ECM glycoproteins	Fibronectin 1	Fibronectin 1 OS=Sus scrofa OX=9823 GN=FN1 PE=1 SV=1	8.109000325	tr|A0A286ZY95|A0A286ZY95_PIG

ECM glycoproteins	Apolipoprotein D	Apolipoprotein D OS=Homo sapiens OX=9606 GN=APOD PE=1 SV=1	38.10000122	sp|P05090|APOD_HUMAN
ECM affiliated proteins	Annexin	Annexin OS=Equus caballus OX=9796 GN=ANXA2 PE=2 SV=1	37.45999932	tr|F6ZI51|F6ZI51_HORSE
ECM affiliated proteins	Annexin	Annexin OS=Sus scrofa OX=9823 GN=ANXA6 PE=2 SV=1	17.38000065	tr|M3VH45|M3VH45_PIG
ECM affiliated proteins	Dermatopontin	Dermatopontin OS=Ovis aries OX=9940 GN=DPT PE=4 SV=1	11.94000021	tr|W5PHI8|W5PHI8_SHEEP
Secreted factors	Protein S100	Protein S100 OS=Pan troglodytes OX=9598 GN=S100A8 PE=3 SV=1	11.82999983	tr|H2Q028|H2Q028_PANTR
Others extracellular components	Azurocidin	Azurocidin OS=Sus scrofa OX=9823 GN=AZU1 PE=1 SV=2	52.85000205	sp|P80015|CAP7_PIG
Others extracellular components	Kappa-casein	Kappa-casein (Fragment) OS=Bos indicus x Bos taurus OX=30522 GN=CSN3 PE=4 SV=1	17.4999997	tr|Q9N273|Q9N273_BOBOX
Others extracellular components	Serum albumin	Serum albumin OS=Homo sapiens OX=9606 PE=2 SV=1	14.77999985	tr|Q56G89|Q56G89_HUMAN
Cellular Components	Caveolin	Caveolin OS=Castor canadensis OX=51338 GN=CAV1 PE=3 SV=1	19.09999996	tr|A0A250XVR7|A0A250XVR7_CASCN
Cellular Components	Caveolin	Caveolin OS=Sus scrofa OX=9823 GN=CAV2 PE=2 SV=1	17.28000045	tr|G8GCE6|G8GCE6_PIG
Cellular Components	cellular enzymes	Histone acetyltransferase (Fragment) OS=Tupaia chinensis OX=246437 GN=TREES_T100014382 PE=3 SV=1	0.775900017	tr|L9JAG0|L9JAG0_TUPCH
Cellular Components	cellular enzymes	Glyceraldehyde-3-phosphate dehydrogenase OS=Homo sapiens OX=9606 GN=HEL-S-162eP PE=2 SV=1	15.21999985	tr|V9HVZ4|V9HVZ4_HUMAN
Cellular Components	cellular enzymes	Phosphoinositide 3-kinase adapter protein 1 (Fragment) OS=Heterocephalus glaber OX=10181 GN=GW7_11923 PE=4 SV=1	2.553999983	tr|G5BEU1|G5BEU1_HETGA
Cellular Components	myosin 11	REVERSED MKIAA0866 protein (Fragment) OS=Mus musculus OX=10090 GN=Myh11 PE=2 SV=1	0.453600008	RRRRRtr|Q69ZX3|Q69ZX3_MOUSE
Cellular Components	Actin	Actin, aortic smooth muscle OS=Cricetulus griseus OX=10029 GN=H671_3g9856 PE=3 SV=1	24.60000068	tr|G3HQY2|G3HQY2_CRIGR
Cellular Components	Ribosomal proteins	OS=Camelus ferus OX=419612 GN=CB1_000878024 PE=4 SV=1	30.98999858	tr|S9Y7C3|S9Y7C3_CAMFR
Cellular Components	Beta-1-syntrophin	Beta-1-syntrophin OS=Fukomys damarensis OX=885580 GN=H920_03321 PE=4 SV=1	3.694999963	tr|A0A091EIA7|A0A091EIA7_FUKDA
Cellular Components	Myeloid-associated differentiation marker	Myeloid-associated differentiation marker tv2 OS=Sus scrofa OX=9823 GN=MYADM PE=2 SV=1	18.32000017	tr|M3UZ75|M3UZ75_PIG
Cellular Components	Junction plakoglobin	Junction plakoglobin OS=Myotis brandtii OX=109478 GN=D623_10015352 PE=4 SV=1	22.82000035	tr|S7NBB7|S7NBB7_MYOBR

Supplementary Table 3. List of peptides found in proteomic analysis of No-DC Endo.

General protein classification	Protein classification	Peptide name	% Cov (Coverage)	Accession
Collagen	Collagen type I	Collagen alpha-1(I) chain (Fragments) OS=Cyclopes didactylus OX=84074 GN=COL1A1 PE=1 SV=1	90.200001	sp|C0HJP1|CO1A1_CYCDI
Collagen	Collagen type I	Collagen alpha-1(I) chain (Fragments) OS=Toxodon sp. OX=1563122 GN=COL1A1 PE=1 SV=1	88.09000254	sp|C0HJP7|CO1A1_TOXSP
Collagen	Collagen type I	Collagen alpha-1(I) chain OS=Castor canadensis OX=51338 GN=COL1A1 PE=4 SV=1	63.22000027	tr|A0A250Y7T0|A0A250Y7T0_CASCN
Collagen	Collagen type I	Collagen alpha-1(I) chain OS=Dipodomys ordii OX=10020 GN=Col1a1 PE=4 SV=1	59.43999887	tr|A0A1S3GXN3|A0A1S3GXN3_DIPOR
Collagen	Collagen type I	Collagen type I alpha 1 chain OS=Felis catus OX=9685 GN=COL1A1 PE=4 SV=2	66.53000116	tr|M3W2F5|M3W2F5_FELCA
Collagen	Collagen type I	Collagen type I alpha 1 chain OS=Sus scrofa OX=9823 GN=COL1A1 PE=1 SV=1	70.05000114	tr|A0A287A1S6|A0A287A1S6_PIG
Collagen	Collagen type I	Collagen type I alpha 2 chain OS=Otolemur garnettii OX=30611 GN=COL1A2 PE=4 SV=1	42.44999886	tr|H0WT85|H0WT85_OTOGA
Collagen	Collagen type I	Collagen alpha-2(I) chain OS=Mammut americanum OX=39053 PE=1 SV=3	57.49999881	sp|P85154|CO1A2_MAMAE

Collagen	Collagen type I	Collagen alpha-2(I) chain OS=Rattus norvegicus OX=10116 GN=Col1a2 PE=1 SV=3	45.26000023	tr|F1LS40|F1LS40_RAT
Collagen	Collagen type III	Collagen alpha-1(III) chain OS=Bos taurus OX=9913 GN=COL3A1 PE=1 SV=1	69.20999885	sp|P04258|CO3A1_BOVIN
Collagen	Collagen type III	Collagen type III alpha 1 chain OS=Equus caballus OX=9796 GN=COL3A1 PE=4 SV=1	37.94000149	tr|F6R528|F6R528_HORSE
Collagen	Collagen type III	Collagen type III alpha 1 chain OS=Mustela putorius furo OX=9669 GN=COL3A1 PE=4 SV=1	41.53999984	tr|M3YLM6|M3YLM6_MUSPF
Collagen	Collagen type III	Collagen type III alpha 1 chain OS=Myotis lucifugus OX=59463 GN=COL3A1 PE=4 SV=1	42.10000038	tr|G1PR85|G1PR85_MYOLU
Collagen	Collagen type IV	Collagen type IV alpha 1 chain OS=Mustela putorius furo OX=9669 GN=COL4A1 PE=3 SV=1	16.36999995	tr|M3YI93|M3YI93_MUSPF
Collagen	Collagen type IV	Collagen type IV alpha 2 chain OS=Sus scrofa OX=9823 GN=COL4A2 PE=1 SV=3	12.72999942	tr|F1RLL9|F1RLL9_PIG
Collagen	Collagen type V	ProCollagen alpha 1(V) OS=Sus scrofa OX=9823 GN=COL5A1 PE=2 SV=1	19.23999935	tr|Q59IP3|Q59IP3_PIG
Collagen	Collagen type V	ProCollagen alpha 2(V) OS=Sus scrofa OX=9823 GN=COL5A2 PE=2 SV=1	27.27999985	tr|Q59IP2|Q59IP2_PIG
Collagen	Collagen type VI	Collagen type VI alpha 1 chain OS=Otolemur garnettii OX=30611 GN=COL6A1 PE=4 SV=1	10.27000025	tr|H0Y0P4|H0Y0P4_OTOGA
Collagen	Collagen type VI	Collagen type VI alpha 2 chain OS=Bos taurus OX=9913 GN=COL6A2 PE=1 SV=1	14.8300007	tr|Q1JQB0|Q1JQB0_BOVIN
Collagen	Collagen type VI	Collagen type VI alpha 3 chain OS=Otolemur garnettii OX=30611 GN=COL6A3 PE=4 SV=1	4.416000098	tr|H0XEJ5|H0XEJ5_OTOGA
Collagen	Collagen type VI	Collagen type VI alpha 3 chain OS=Ovis aries OX=9940 GN=COL6A3 PE=4 SV=1	5.040999874	tr|W5QCP9|W5QCP9_SHEEP
Collagen	Collagen type VI	Collagen type VI alpha 3 chain OS=Sus scrofa OX=9823 GN=COL6A3 PE=1 SV=1	10.22000015	tr|A0A287BLM4|A0A287BLM4_PIG
Collagen	Collagen type VI	Collagen type VI alpha 3 chain OS=Sus scrofa OX=9823 GN=COL6A3 PE=1 SV=2	4.397	tr|I3LUR7|I3LUR7_PIG
ECM glycoproteins	Laminin	Laminin subunit alpha 5 OS=Sus scrofa OX=9823 GN=LAMA5 PE=4 SV=1	2.474999987	tr|A0A287AEH1|A0A287AEH1_PIG
ECM glycoproteins	Laminin	Laminin, alpha 5 OS=Pan troglodytes OX=9598 GN=LAMA5 PE=2 SV=1	1.813000068	tr|K7D2I3|K7D2I3_PANTR
ECM glycoproteins	Laminin	Laminin subunit beta 1 OS=Sus scrofa OX=9823 GN=LAMB1 PE=1 SV=3	2.50599999	tr|F1SAE9|F1SAE9_PIG
ECM glycoproteins	Laminin	Laminin subunit beta 2 OS=Sus scrofa OX=9823 GN=LAMB2 PE=1 SV=3	7.274000347	tr|F1SPT5|F1SPT5_PIG
ECM glycoproteins	Fibrillin	Fibrillin-1 OS=Sus scrofa OX=9823 GN=FBN1 PE=1 SV=3	17.82999933	tr|F1SN67|F1SN67_PIG
ECM glycoproteins	Fibronectin 1	Fibronectin 1 OS=Sus scrofa OX=9823 GN=FN1 PE=1 SV=1	12.26999983	tr|A0A286ZY95|A0A286ZY95_PIG
ECM glycoproteins	Nidogen 1	Nidogen 1 OS=Sus scrofa OX=9823 GN=NID1 PE=1 SV=3	6.768999994	tr|F1RGY5|F1RGY5_PIG
ECM glycoproteins	von Willebrand factor	von Willebrand factor OS=Sus scrofa OX=9823 GN=VWF PE=1 SV=2	4.417999834	tr|K7GNN0|K7GNN0_PIG
ECM glycoproteins	Apolipoprotein D	Apolipoprotein D OS=Ovis aries OX=9940 GN=APOD PE=3 SV=1	13.15000057	tr|W5QGP4|W5QGP4_SHEEP
ECM glycoproteins	Adiponectin	30 kDa adipocyte complement-related protein OS=Rattus norvegicus OX=10116 GN=Adipoq PE=1 SV=1	24.17999953	tr|Q8K3R4|Q8K3R4_RAT
ECM regulators	Serpin Family	Leukocyte elastase inhibitor OS=Sus scrofa OX=9823 GN=SERPINB1 PE=1 SV=1	47.62000144	tr|F2Z5B1|F2Z5B1_PIG
ECM regulators	Serpin Family	Serpin family B member 6 OS=Sus scrofa OX=9823 GN=SERPINB6 PE=1 SV=2	25.40000081	tr|I3LCP8|I3LCP8_PIG
ECM regulators	Serpin Family	Serpin family F member 2 OS=Sus scrofa OX=9823 GN=SERPINF2 PE=1 SV=1	10.30000001	tr|A0A287B9B3|A0A287B9B3_PIG
ECM regulators	Serpin Family	Alpha-1-antichymotrypsin 2 OS=Sus scrofa OX=9823 GN=SERPINA3-2 PE=3 SV=1	12.04999983	tr|Q9GMA6|Q9GMA6_PIG
ECM regulators	Serpin Family	Alpha-1-antitrypsin OS=Sus scrofa OX=9823 GN=SERPINA1 PE=3 SV=2	15.0000006	tr|F1SCF0|F1SCF0_PIG
ECM regulators	Cathepsin	Cathepsin B OS=Sus scrofa OX=9823 GN=CTSB PE=1 SV=1	21.2500006	tr|A0A287BF94|A0A287BF94_PIG
ECM regulators	Cathepsin	Cathepsin D protein (Fragment) OS=Sus scrofa OX=9823 PE=2 SV=1	23.0399996	tr|Q5MJE5|Q5MJE5_PIG
ECM regulators	Cathepsin	Cathepsin K OS=Sus scrofa OX=9823 GN=CTSS PE=1 SV=2	9.063000232	tr|F1SS93|F1SS93_PIG
ECM regulators	Cathepsin	Cathepsin Z OS=Sus scrofa OX=9823 GN=CTSZ PE=1 SV=1	11.18000001	tr|A5GFX7|A5GFX7_PIG
ECM affiliated proteins	Annexin	Annexin OS=Sus scrofa OX=9823 GN=ANXA1 PE=1 SV=2	22.22000062	tr|K7GLE1|K7GLE1_PIG
ECM affiliated proteins	Annexin	Annexin OS=Sus scrofa OX=9823 GN=ANXA2 PE=1 SV=1	18.84000003	tr|A0A286ZJV6|A0A286ZJV6_PIG
ECM affiliated proteins	Mucin	Mucin 5AC, oligomeric mucus/gel-forming OS=Sus scrofa OX=9823 GN=MUC5AC PE=1 SV=1	1.121000014	tr|A0A287ANG4|A0A287ANG4_PIG
Secreted factors	Protein S100	Protein S100 OS=Pan troglodytes OX=9598 GN=S100A8 PE=3 SV=1	37.63000071	tr|H2Q028|H2Q028_PANTR
Others extracellular components	Serum albumin	Serum albumin (Fragment) OS=Homo sapiens OX=9606 GN=ALB PE=1 SV=1	19.59999949	tr|H0YA55|H0YA55_HUMAN
Others extracellular	Serum albumin	Serum albumin OS=Sus scrofa OX=9823 GN=ALB PE=1 SV=1	44.47999895	tr|F1RUN2|F1RUN2_PIG

components				
Cellular components	cellular enzymes	Glyceraldehyde-3-phosphate dehydrogenase OS=Homo sapiens OX=9606 GN=HEL-S-162eP PE=2 SV=1	27.1600008	tr|V9HVZ4|V9HVZ4_HUMAN
Cellular components	cellular enzymes	Cytidine deaminase OS=Bos mutus OX=72004 GN=M91_09666 PE=3 SV=1	23.97000045	tr|L8I1I7|L8I1I7_9CETA
Cellular components	cellular enzymes	D-aminoacyl-tRNA deacylase OS=Sus scrofa OX=9823 GN=DTD1 PE=1 SV=2	16.26999974	tr|F1SBH1|F1SBH1_PIG
Cellular components	cellular enzymes	Dicarbonyl and L-xylulose reductase OS=Sus scrofa OX=9823 GN=DCXR PE=4 SV=1	23.77000004	tr|A0A286ZQ44|A0A286ZQ44_PIG
Cellular components	cellular enzymes	Glucosidase alpha, acid OS=Sus scrofa OX=9823 GN=GAA PE=1 SV=2	7.427000254	tr|I3LQL8|I3LQL8_PIG
Cellular components	cellular enzymes	Glutathione peroxidase OS=Sus scrofa OX=9823 GN=GPX3 PE=1 SV=1	23.99999946	tr|A0A287AIJ3|A0A287AIJ3_PIG
Cellular components	cellular enzymes	GMP reductase OS=Ovis aries OX=9940 GN=GMPR2 PE=3 SV=1	24.78999943	tr|W5QCY6|W5QCY6_SHEEP
Cellular components	cellular enzymes	GMP synthase [glutamine-hydrolyzing] OS=Cricetulus griseus OX=10029 GN=I79_004230 PE=4 SV=1	4.055000097	tr|G3H229|G3H229_CRIGR
Cellular components	cellular enzymes	Thioredoxin-dependent peroxide reductase, mitochondrial OS=Bos mutus OX=72004 GN=M91_10467 PE=4 SV=1	16.73000008	tr|L8I536|L8I536_9CETA
Cellular components	cellular enzymes	Maltase-glucoamylase OS=Sus scrofa OX=9823 GN=MGAM PE=1 SV=1	12.48999983	tr|A0A287A042|A0A287A042_PIG
Cellular components	cellular enzymes	N-acetylglucosamine-6-sulfatase OS=Sus scrofa OX=9823 GN=GNS PE=1 SV=1	8.696000278	tr|K9IVU5|K9IVU5_PIG
Cellular components	cellular enzymes	N-acylethanolamine acid amidase OS=Sus scrofa OX=9823 GN=NAAA PE=1 SV=2	16.57000035	tr|F1RYU7|F1RYU7_PIG
Cellular components	cellular enzymes	N-acylsphingosine amidohydrolase 1 OS=Sus scrofa OX=9823 GN=ASAH1 PE=1 SV=3	24.05000031	tr|F1SES5|F1SES5_PIG
Cellular components	cellular enzymes	Palmitoyl-protein thioesterase 1 OS=Ovis aries OX=9940 GN=PPT1 PE=4 SV=1	19.86999959	tr|W5QG24|W5QG24_SHEEP
Cellular components	cellular enzymes	Peptidyl-prolyl cis-trans isomerase OS=Cricetulus griseus OX=10029 GN=I79_005402 PE=3 SV=1	9.72200036	tr|G3H533|G3H533_CRIGR
Cellular components	cellular enzymes	Phospholipase B-like OS=Sus scrofa OX=9823 GN=PLBD2 PE=1 SV=3	4.244000092	tr|F1RKC7|F1RKC7_PIG
Cellular components	cellular enzymes	Phospholipase D3 isoform 7-like protein OS=Camelus ferus OX=419612 GN=CB1_000338009 PE=4 SV=1	6.395000219	tr|S9YPW0|S9YPW0_CAMFR
Cellular components	cellular enzymes	Prostaglandin D synthase (Fragment) OS=Sus scrofa OX=9823 GN=pgds PE=2 SV=1	30.93000054	tr|Q765P8|Q765P8_PIG
Cellular components	cellular enzymes	Superoxide dismutase [Cu-Zn] OS=Sus scrofa OX=9823 GN=SOD1 PE=2 SV=1	39.21999931	tr|D9D839|D9D839_PIG
Cellular components	cellular enzymes	Transaldolase OS=Sus scrofa OX=9823 GN=TALDO1 PE=1 SV=3	10.98000035	tr|F1RYY6|F1RYY6_PIG
Cellular components	cellular enzymes	3-hydroxybutyrate dehydrogenase type 2 OS=Fukomys damarensis OX=885580 GN=H920_12791 PE=3 SV=1	10.98000035	tr|A0A091D5V5|A0A091D5V5_FUKDA
Cellular components	cellular enzymes	Histone acetyltransferase (Fragment) OS=Tupaia chinensis OX=246437 GN=TREES_T100014382 PE=3 SV=1	4.518000036	tr|L9JAG0|L9JAG0_TUPCH
Cellular components	cellular enzymes	Ribonuclease 4 OS=Sus scrofa OX=9823 GN=RNASE4 PE=1 SV=3	35.3700012	sp|P15468|RNAS4_PIG
Cellular components	Caveolin	Caveolin OS=Sus scrofa OX=9823 GN=CAV2 PE=2 SV=1	17.28000045	tr|G8GCE6|G8GCE6_PIG
Cellular components	Myosin	Myosin heavy chain 9 OS=Felis catus OX=9685 GN=MYH9 PE=3 SV=2	8.27300027	tr|M3VW11|M3VW11_FELCA
Cellular components	Myosin	Myosin-10 isoform 2 OS=Callithrix jacchus OX=9483 GN=MYH10 PE=2 SV=1	6.123000011	tr|U3F1R2|U3F1R2_CALJA
Cellular components	Myosin	Myosin-11 OS=Pteropus alecto OX=9402 GN=PAL_GLEAN10009738 PE=3 SV=1	9.788999707	tr|L5KQB6|L5KQB6_PTEAL
Cellular components	Tropomiosin	Tropomyosin 1 (Alpha), isoform CRA_f OS=Homo sapiens OX=9606 GN=TPM1 PE=1 SV=1	52.14999914	tr|Q6ZN40|Q6ZN40_HUMAN
Cellular components	Tropomiosin	Tropomyosin alpha-1 chain isoform 3 OS=Callithrix jacchus OX=9483 GN=TPM1 PE=2 SV=1	60.21000147	tr|L5K201|L5K201_PTEAL
Cellular components	Tropomiosin	Tropomyosin alpha-3 chain isoform 2 OS=Macaca mulatta OX=9544 GN=TPM3 PE=2 SV=1	68.54000092	tr|U3E2P5|U3E2P5_CALJA
Cellular components	Tropomiosin	Tropomyosin alpha-3 chain OS=Pteropus alecto OX=9402 GN=PAL_GLEAN10023407 PE=3 SV=1	48.51999879	tr|H9YZ58|H9YZ58_MACMU
Cellular components	Tropomiosin	Tropomyosin 2 OS=Ovis aries OX=9940 GN=TPM2 PE=3 SV=1	57.74999857	tr|W5PQL4|W5PQL4_SHEEP
Cellular components	Tropomiosin	Epididymis secretory protein Li 108 OS=Homo sapiens OX=9606 GN=HEL-S-108 PE=2 SV=1	74.59999919	tr|V9HW56|V9HW56_HUMAN
Cellular components	Histones	Heterogeneous nuclear ribonucleoprotein U OS=Ovis aries OX=9940 GN=HNRNPU PE=4 SV=1	6.612999737	tr|W5P4I9|W5P4I9_SHEEP
Cellular components	Histones	Heterogeneous nuclear ribonucleoprotein H3 OS=Loxodonta africana OX=9785 GN=HNRNPH3 PE=4 SV=1	16.14000052	tr|G3TAE6|G3TAE6_LOXAF
Cellular components	Histones	Core histone macro-H2A OS=Sarcophilus harrisii OX=9305 GN=H2AFY PE=4 SV=1	11.02000028	tr|G3W5X9|G3W5X9_SARHA
Cellular components	Others	Proliferating cell nuclear antigen OS=Ovis aries OX=9940 GN=PCNA PE=3 SV=1	13.1099999	tr|W5Q6P4|W5Q6P4_SHEEP

Cellular components	Others	SEC22 homolog B, vesicle trafficking protein (gene/pseudogene) OS=Ovis aries OX=9940 GN=SEC22B PE=3 SV=1	6.421999633	tr|W5QGX9|W5QGX9_SHEEP
Cellular components	Others	Selenium binding protein 1 OS=Sus scrofa OX=9823 GN=SELENBP1 PE=1 SV=2	17.03000069	tr|F1ST01|F1ST01_PIG
Cellular components	Others	Solute carrier family 44 member 1 OS=Ovis aries OX=9940 GN=SLC44A1 PE=3 SV=1	4.715000093	tr|W5PBL5|W5PBL5_SHEEP
Cellular components	Others	Spectrin beta chain OS=Ovis aries OX=9940 GN=SPTBN1 PE=3 SV=1	3.088999912	tr|W5NZX9|W5NZX9_SHEEP
Cellular components	Others	transcriptional activator protein Pur-alpha OS=Dipodomys ordii OX=10020 GN=Pura PE=4 SV=1	13.84000033	tr|A0A1S3FI87|A0A1S3FI87_DIPOR
Cellular components	Others	Caveolae associated protein 1 OS=Chlorocebus sabaeus OX=60711 GN=CAVIN1 PE=4 SV=1	18.62999946	tr|A0A0D9S1T8|A0A0D9S1T8_CHLSB
Cellular components	Others	CDGSH iron sulfur domain 2 OS=Ovis aries OX=9940 GN=CISD2 PE=4 SV=1	13.72999996	tr|W5PRB3|W5PRB3_SHEEP
Cellular components	Others	Filamin A OS=Sus scrofa OX=9823 GN=FLNA PE=4 SV=1	2.410000004	tr|A0A287B242|A0A287B242_PIG
Cellular components	Others	Filamin C OS=Ovis aries OX=9940 GN=FLNC PE=4 SV=1	6.216000021	tr|W5NZK9|W5NZK9_SHEEP
Cellular components	Others	Ferritin OS=Camelus ferus OX=419612 GN=CB1_000743158 PE=3 SV=1	13.24999928	tr|S9WS69|S9WS69_CAMFR
Cellular components	Others	Hemoglobin subunit beta OS=Sus scrofa OX=9823 GN=HBB PE=3 SV=1	45.57999969	tr|F1RII7|F1RII7_PIG
Cellular components	Others	Voltage dependent anion channel 1 OS=Ictidomys tridecemlineatus OX=43179 GN=VDAC1 PE=4 SV=2	31.85000122	tr|I3MAD1|I3MAD1_ICTTR
Cellular components	Others	voltage-dependent anion-selective channel protein 3 OS=Dipodomys ordii OX=10020 GN=Vdac3 PE=4 SV=1	29.67999876	tr|A0A1S3G0R9|A0A1S3G0R9_DIPOR
Cellular components	Others	Polyubiquitin-C OS=Macaca fascicularis OX=9541 GN=EGM_07462 PE=4 SV=1	31.36999905	tr|G7PTR1|G7PTR1_MACFA
Cellular components	Others	Prelamin-A/C OS=Sus scrofa OX=9823 GN=LMNA PE=1 SV=1	12.5	tr|F1RLQ2|F1RLQ2_PIG
Cellular components	Ribosomal proteins	Ribosomal protein S3 OS=Ovis aries OX=9940 GN=RPS3 PE=3 SV=1	17.08000004	tr|W5PPH6|W5PPH6_SHEEP
Cellular components	Ribosomal proteins	40S ribosomal protein S3a OS=Ovis aries OX=9940 GN=RPS3A PE=3 SV=1	24.24000055	tr|W5QG75|W5QG75_SHEEP
Cellular components	Ribosomal proteins	40S ribosomal protein SA OS=Myotis brandtii OX=109478 GN=RPSA PE=3 SV=1	29.19000089	tr|S7P8J3|S7P8J3_MYOBR
Cellular components	Ribosomal proteins	60S ribosomal protein L18 OS=Camelus ferus OX=419612 GN=CB1_007371005 PE=4 SV=1	19.14999932	tr|S9W5U7|S9W5U7_CAMFR
Cellular components	Ribosomal proteins	60S ribosomal protein L22 OS=Pteropus alecto OX=9402 GN=PAL_GLEAN10013085 PE=4 SV=1	11.21999994	tr|L5KGY7|L5KGY7_PTEAL
Cellular components	Inter-alpha-trypsin inhibitor	Inter-alpha-trypsin inhibitor heavy chain H1 OS=Sus scrofa OX=9823 GN=ITIH1 PE=1 SV=3	10.09000018	tr|F1SH96|F1SH96_PIG
Cellular components	Junction plakoglobin	Junction plakoglobin OS=Myotis brandtii OX=109478 GN=D623_10015352 PE=4 SV=1	22.14999944	tr|S7NBB7|S7NBB7_MYOBR
Cellular components	Legumain	Legumain OS=Sus scrofa OX=9823 GN=LGMN PE=1 SV=2	2.740000002	tr|I3LKM9|I3LKM9_PIG
Cellular components	Thioredoxin	Thioredoxin OS=Sus scrofa OX=9823 GN=TRX1 PE=2 SV=1	46.66999876	tr|H6TBN0|H6TBN0_PIG
Cellular components	peroxiredoxin	Epididymis secretory sperm binding protein Li 97n OS=Homo sapiens OX=9606 GN=HEL-S-97n PE=2 SV=1	15.12999982	tr|V9HW63|V9HW63_HUMAN
Ig	IgA	IgA heavy chian constant region (Fragment) OS=Sus scrofa OX=9823 GN=IGHA PE=4 SV=1	17.2999993	tr|K7ZRK0|K7ZRK0_PIG
Ig	IgG	IgG H chain OS=Homo sapiens OX=9606 PE=2 SV=1	20.09000033	tr|S6BAP0|S6BAP0_HUMAN
Ig	IgG	IgG heavy chain OS=Sus scrofa OX=9823 GN=IGHG PE=2 SV=1	49.88999963	tr|L8B0R9|L8B0R9_PIG
Ig	IgG	IgG heavy chain OS=Sus scrofa OX=9823 GN=IGHG PE=2 SV=1	55.22000194	tr|L8B0W0|L8B0W0_PIG
Ig	IgG	IgG heavy chain OS=Sus scrofa OX=9823 GN=IGHG PE=2 SV=1	45.35999894	tr|L8B0V6|L8B0V6_PIG
Ig	IgG	IgG heavy chain OS=Sus scrofa OX=9823 GN=IGHG PE=2 SV=1	38.49000037	tr|L8B139|L8B139_PIG
Ig	IgG	IgG heavy chain OS=Sus scrofa OX=9823 GN=IGHG PE=2 SV=1	38.17000091	tr|L8B0S2|L8B0S2_PIG
Ig	IgG	IgG heavy chain OS=Sus scrofa OX=9823 GN=IGHG PE=2 SV=1	45.9100008	tr|L8B180|L8B180_PIG
Ig	IgG	IgG heavy chain OS=Sus scrofa OX=9823 GN=IGHG PE=2 SV=1	39.48000073	tr|L8AXL3|L8AXL3_PIG
Ig	IgG	IgG heavy chain OS=Sus scrofa OX=9823 GN=IGHG PE=2 SV=1	40.43000042	tr|L8B0U8|L8B0U8_PIG
Ig	IgG	IgG heavy chain OS=Sus scrofa OX=9823 GN=IGHG PE=2 SV=1	27.75000036	tr|L8AXL9|L8AXL9_PIG
Ig	IgG	IgG heavy chain OS=Sus scrofa OX=9823 GN=IGHG PE=2 SV=1	49.03999865	tr|L8B173|L8B173_PIG
Ig	IgG	IgG heavy chian constant region (Fragment) OS=Sus scrofa OX=9823 GN=IGHG5-1 PE=4 SV=1	31.58000112	tr|K7ZPU8|K7ZPU8_PIG

Ig	IgG	IgG L chain OS=Homo sapiens OX=9606 PE=2 SV=1	8.64899978	tr|S6B294|S6B294_HUMAN
MHC class II antigen	MHC class II antigen	MHC class II antigen OS=Sus scrofa OX=9823 GN=SLA-DQB PE=2 SV=1	20.69000006	tr|Q8SPA1|Q8SPA1_PIG
MHC class II antigen	MHC class II antigen	MHC class II antigen OS=Sus scrofa OX=9823 GN=SLA-DRA PE=2 SV=1	6.746000051	tr|Q860P1|Q860P1_PIG
MHC class II antigen	MHC class II antigen	MHC class II antigen OS=Sus scrofa OX=9823 GN=SLA-DRB1 PE=2 SV=1	16.92000031	tr|B1A9N6|B1A9N6_PIG

Supplementary Table 4. Matrisome profile of EndoECM, NO-DC Endo, and MyoECM.

		PROTEIN TYPE	EndoECM	NO-DC Endo	MyoECM	GO FUNCTION RELATED WITH EXTRACELLULAR MATRIX PROCESS
CORE MATRISOME	COLLAGENS	**Collagen type I**	■	■	■	ECM structural constituent; identical protein binding; platelet-derived growth factor (PDGF) binding.
		**Collagen type III**	■	■	■	ECM structural constituent conferring tensile strength; integrin binding; PDGF binding.
		**Collagen type IV**		■		ECM structural constituent conferring tensile strength; protein binding; PDGF binding.
		**Collagen type V**	■	■	■	ECM structural constituent conferring tensile strength; integrin binding; heparin binding; proteoglycan binding; PDGF binding.
		**Collagen type VI**	■	■	■	ECM structural constituent conferring tensile strength.
		**Collagen type XII**			■	ECM structural constituent conferring tensile strength.
	ECM GLYCOPROTEINS	**Adiponectin**		■		ECM structural constituent; protein binding; sialic acid binding; protein homodimerization activity.
		**Apolipoprotein D**		■	■	Cholesterol binding.
		**Dermatopontin**	■		■	Collagen fibril organization; cell adhesion.
		**Fibrillin-1**	■	■	■	ECM structural constituent; integrin binding; hormone activity; heparin binding; ECM constituent conferring elasticity.
		**Fibrillin-2**			■	ECM structural constituent; protein binding; Calcium ion binding; ECM constituent conferring elasticity.
		**Fibrinogen**	■			Cell adhesion molecule binding.
		**Fibronectin 1**	■	■	■	ECM structural constituent; integrin binding; collagen binding; heparin binding; proteoglycan binding.
		**Laminin**	■	■	■	ECM structural constituent; integrin binding; structural molecule activity.
		**Nidogen 1**		■		ECM structural constituent; collagen binding; laminin binding; proteoglycan binding.
		**Von Willebrand factor**	■	■		Integrin binding; collagen binding; chaperone binding.
SOME ASSOC IATED	REGU LATO	**Alpha-1-antichymotrypsin 2 (Serpin Family)**		■		Serine-type endopeptidase inhibitor activity.

		**Alpha-1-antitrypsin (Serpin Family)**				Serine-type endopeptidase inhibitor activity.
		**Cathepsin B**				Collagen binding; proteoglycan binding.
		**Cathepsin D**				Aspartic-type endopeptidase activity.
		**Cathepsin S**				Fibronectin binding; collagen binding; laminin binding; proteoglycan binding.
		**Cathepsin Z**				Cysteine-type endopeptidase activity.
		**Leukocyte elastase inhibitor (Serpin Family)**				Serine-type endopeptidase inhibitor activity.
		**Serpin family B member 6 (Serpin Family)**				Protease binding; serine-type endopeptidase inhibitor activity.
		**Serpin family F member 2 (Serpin Family)**				Endopeptidase inhibitor activity; protein binding; protein homodimerization activity.
	ECM-AFFILIATED PROTEINS	**Annexin**				ECM structural constituent; phosphatidylserine binding; actin binding; S100 protein binding; cadherin binding involved in cell-cell adhesion.
		**Mucin**				Extracellular matrix structural constituent.
	SECRETED FACTORS	**Protein S100**				Calcium ion binding; microtubule binding; zinc ion binding; Toll-like receptor 4 binding; arachidonic acid binding; RAGE receptor binding.
OTHER EXTRACELLULAR PROTEINS		**Azurocidin**				Heparin binding; heparan sulfate proteoglycan binding.
		**Extracellular tyrosine-protein kinase**				Protein kinase activity; non-membrane spanning protein tyrosine kinase activity.
		**Kappa-casein**				Protein binding.
		**Serum albumin**				Fatty acid binding; oxygen binding; metal ion binding; chaperone binding.

The datasets presented in this study can be found in Dryad Digital Repository:

López-Martínez, Sara et al. (2021), LC-MS/MS Proteomic data of EndoECM, MyoECM and No-DC Endo, Dryad, Dataset, https://doi.org/10.5061/dryad.vdncjsxsv

References

Shevchenko, A., Jensen, O. N., Podtelejnikov, A. V., Sagliocco, F., Wilm, M., Vorm, O., et al. (1996). Linking genome and proteome by mass spectrometry: Large-scale identification of yeast

proteins from two dimensional gels. in *Proceedings of the National Academy of Sciences of the United States of America*, 14440–14445. doi:10.1073/pnas.93.25.14440.

Shilov, I. V., Seymourt, S. L., Patel, A. A., Loboda, A., Tang, W. H., Keating, S. P., et al. (2007). The paragon algorithm, a next generation search engine that uses sequence temperature values sequence temperature values and feature probabilities to identify peptides from tandem mass spectra. *Mol. Cell. Proteomics* 6, 1638–1655. doi:10.1074/mcp.T600050-MCP200.
