# Supplementary figures and images for: A Natural Xenogeneic Endometrial Extracellular Matrix Hydrogel Toward Improving Current Human in vitro Models and Future in vivo Applications
Source: Front Bioeng Biotechnol. 2021 Mar 5;9:639688. doi: 10.3389/fbioe.2021.639688 (PMC7973233; doi:10.3389/fbioe.2021.639688)

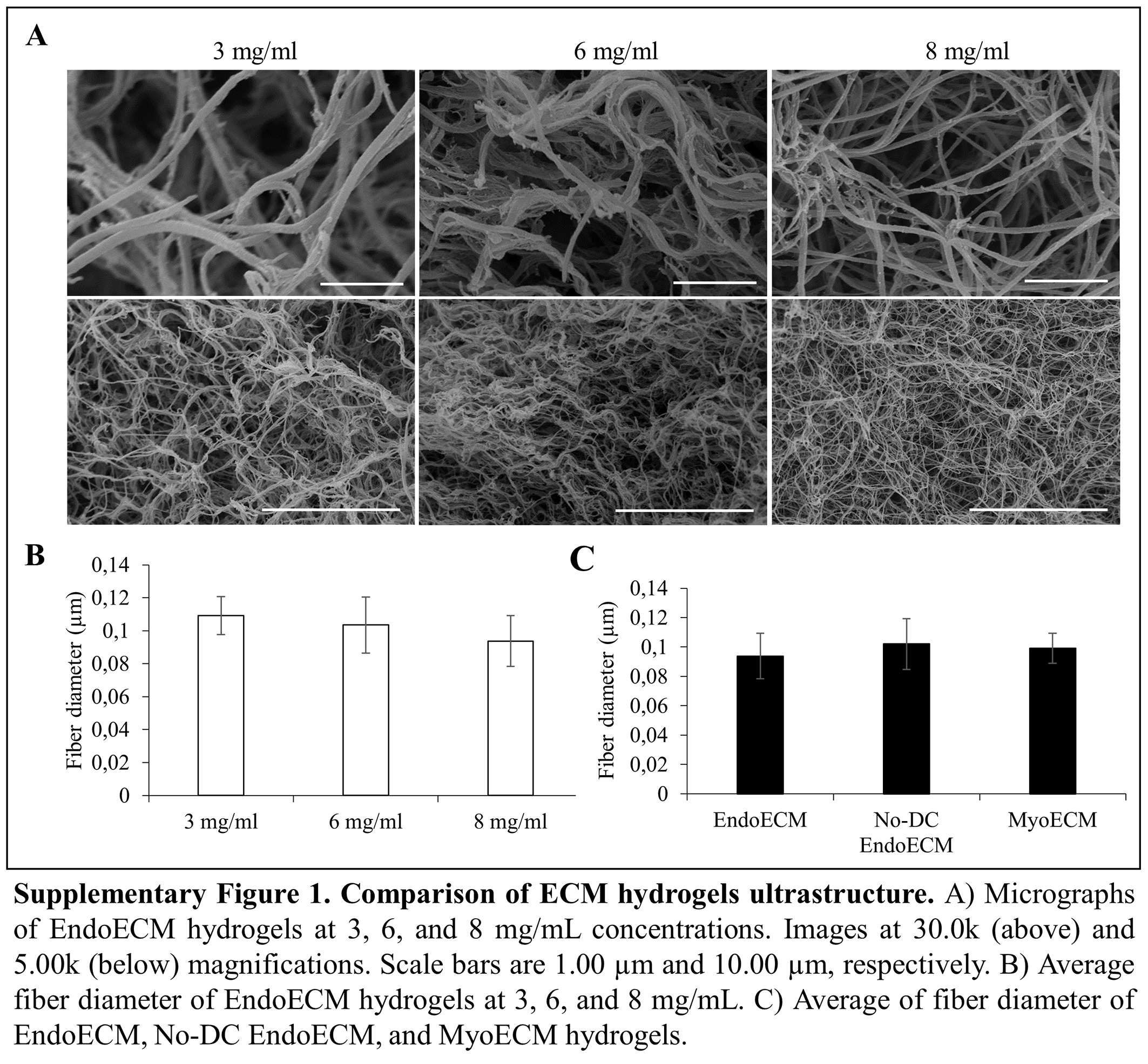

Supplement: Supplementary file 1 [file Data_Sheet_1.zip › SUPPLEMENTARY FIGURE 1.tif]

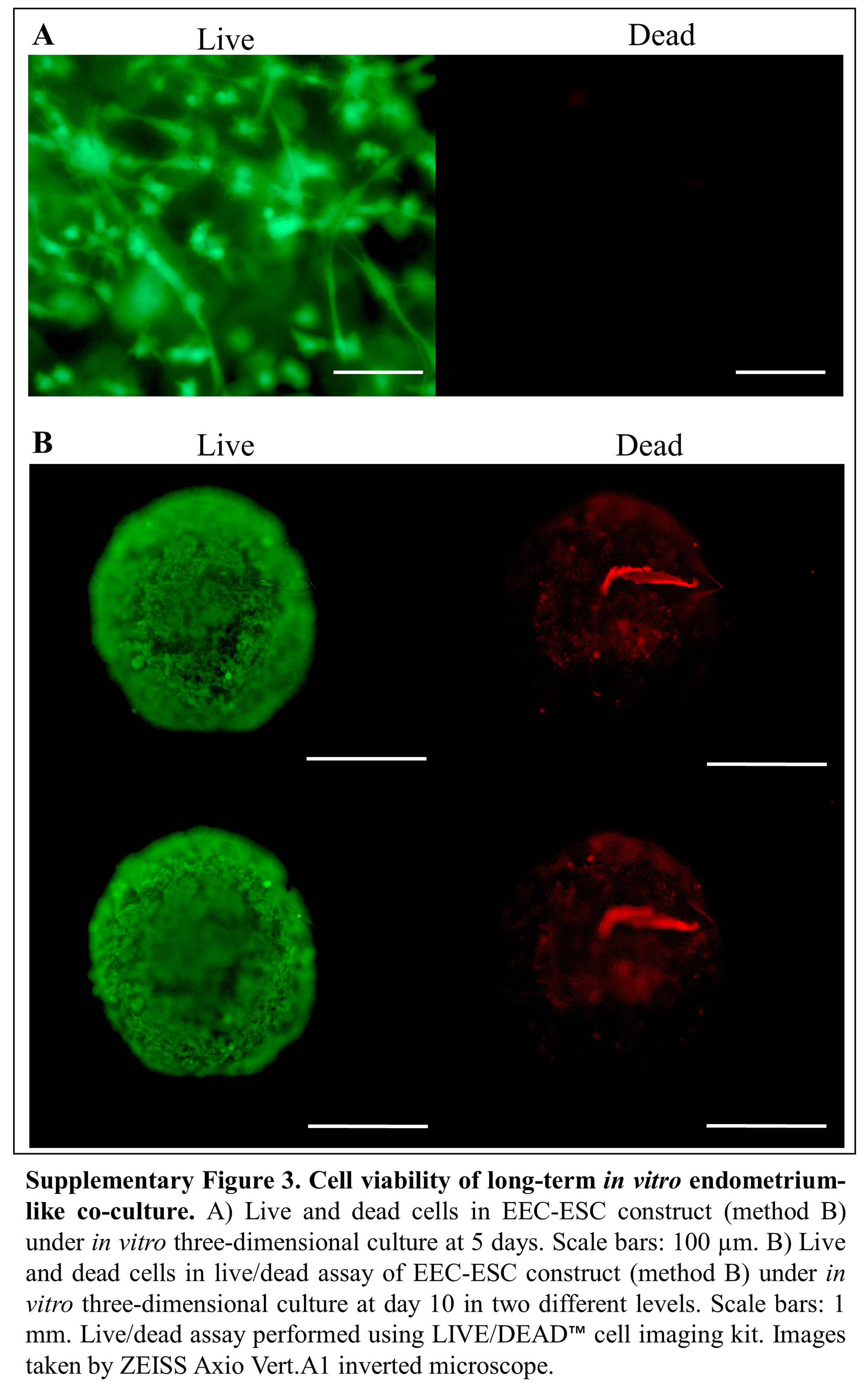

Supplement: Supplementary file 1 [file Data_Sheet_1.zip › SUPPLEMENTARY FIGURE 3.tif]

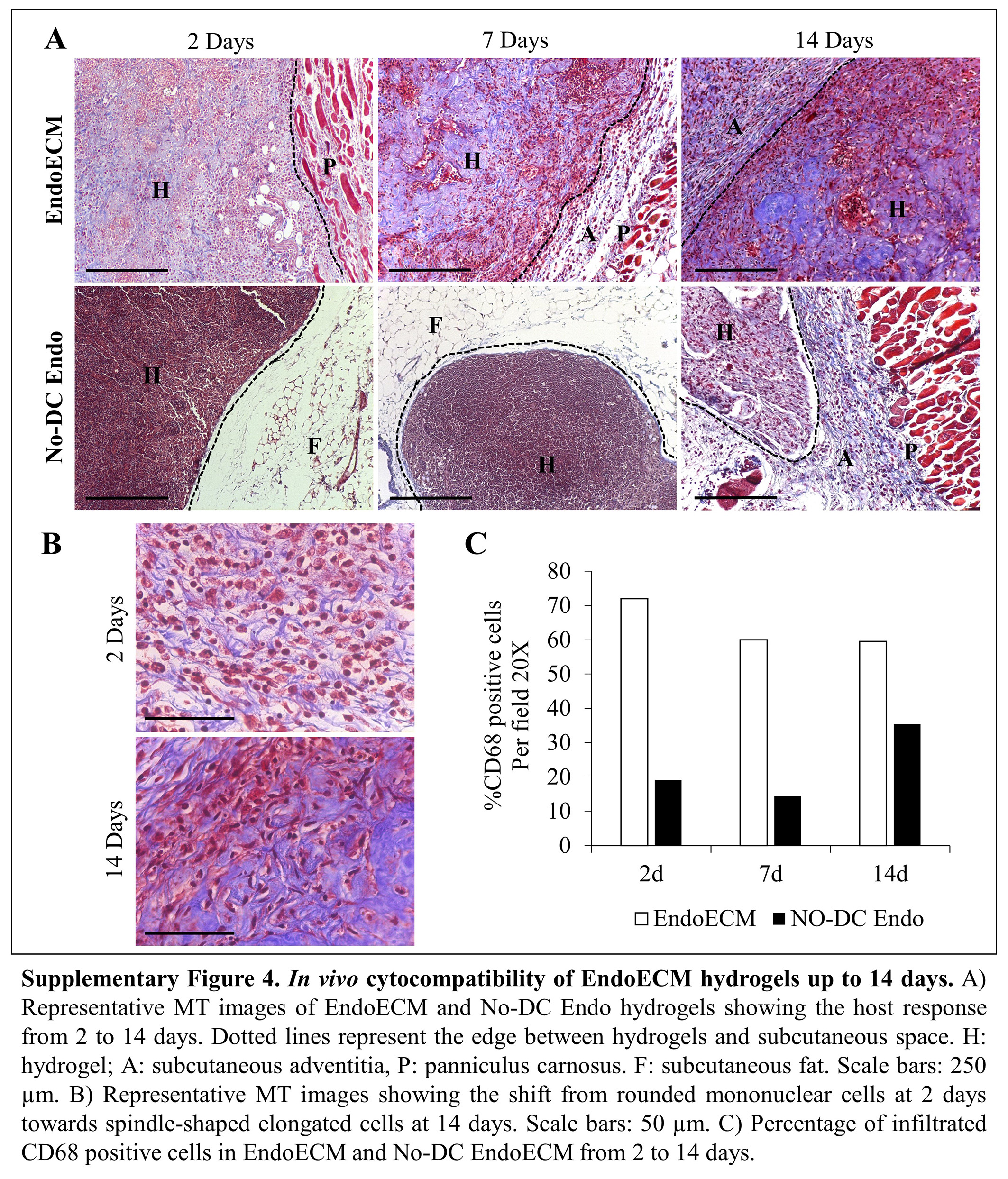

Supplement: Supplementary file 1 [file Data_Sheet_1.zip › SUPPLEMENTARY FIGURE 4.jpg]

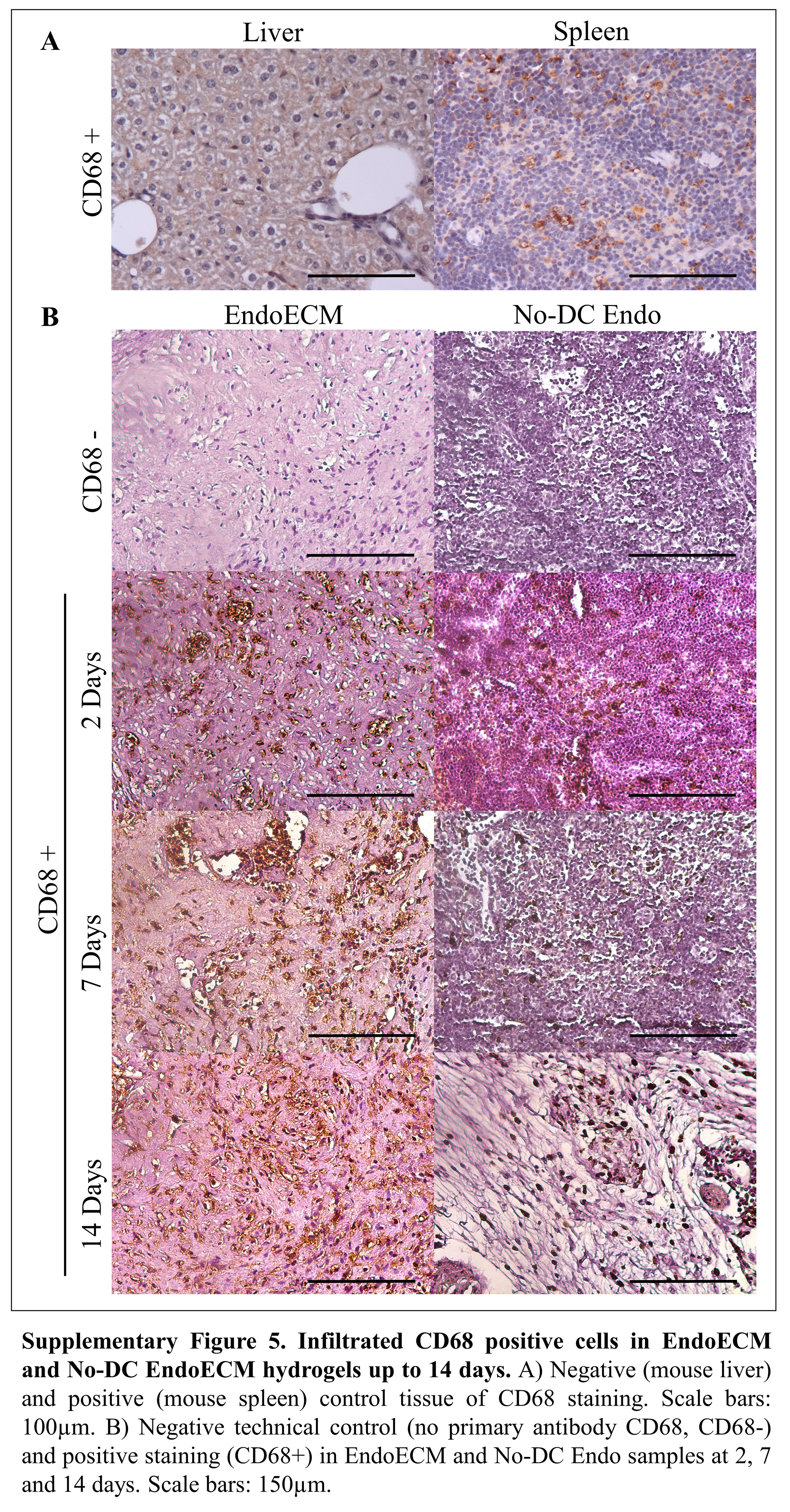

Supplement: Supplementary file 1 [file Data_Sheet_1.zip › SUPPLEMENTARY FIGURE 5.jpg]

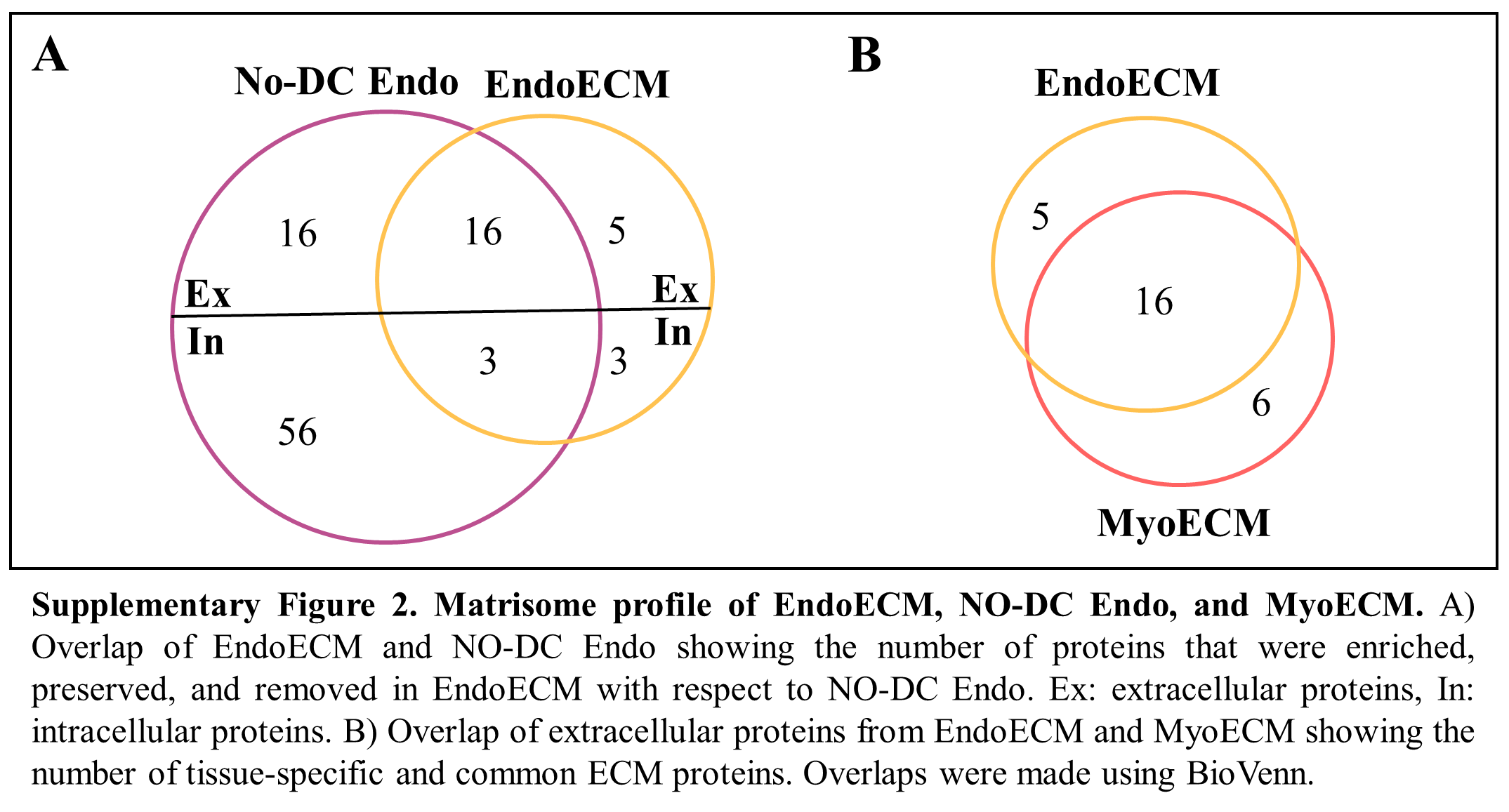

Supplement: Supplementary file 1 [file Data_Sheet_1.zip › SUPPLEMENTARY_FIGURE_2.tif]
